# Maternal control of suspensor programmed cell death via gibberellin signaling

**DOI:** 10.1038/s41467-019-11476-3

**Published:** 2019-08-02

**Authors:** Ce Shi, Pan Luo, Yu-Ting Du, Hong Chen, Xiaorong Huang, Tian-He Cheng, An Luo, Hong-Ju Li, Wei-Cai Yang, Peng Zhao, Meng-Xiang Sun

**Affiliations:** 1 State Key Laboratory of Hybrid Rice, College of Life Sciences, Wuhan University, 430072 Wuhan, China; 2 State Key Laboratory of Molecular and Developmental Biology, Institute of Genetics and Developmental Biology, Chinese Academy of Sciences, 100101 Beijing, China

**Keywords:** Plant embryogenesis, Cell signalling, Seed development

## Abstract

Plant embryos are generated and develop in a stable and well-protected microenvironment surrounded by maternal tissue, which is vital for embryogenesis. However, the signaling mechanisms responsible for maternal tissue-to-proembryo communication are not well understood. Here, we report a pathway for maternal tissue-to-proembryo communication. We identify a DELLA protein, NtCRF1 (*NtCYS* regulative factor 1), which regulates suspensor programmed cell death (PCD). NtCRF1 can bind to the promoter of *NtCYS* and regulate the suspensor PCD-switch module NtCYS-NtCP14 in response to gibberellin (GA). We confirm that GA_4_, as a primary signal triggering suspensor PCD, is generated in the micropylar endothelium by the transient activation of *NtGA3oxs* in the maternal tissue. Thus, we propose that GA is a maternal-to-proembryo communication signal that is decoded in the proembryo by a GID1-CRF1-CYS-CP14 signaling cascade. Using this mode of communication, maternal tissue precisely controls the embryonic suspensor PCD and is able to nurse the proembryo in a stage-dependent manner.

## Abstract (web summary)

Plant embryos are connected to maternal tissue by a filamentous suspensor, analogous to an umbilical cord, but which is severed during early embryo development. Here, Shi et al. show that gibberellins can trigger suspensor cell death in tobacco via a DELLA protein that regulates expression of cell death factors.

## Introduction

Both animal and plant embryos are generated and developed in a stable and well-protected microenvironment, surrounded by maternal tissue. In animals, the maternal tissue impacts the embryonic development through various signals^1–3^. Recent work on the transcriptome of cells at the maternal-fetal interface in humans has shed new light on the mechanisms underlying maternal-fetal communication^4^. In plants, it is also reported that the maternal tissue, and in particular the seed coat, plays a critical role in embryonic development^5–7^, indicating interaction between maternal tissue and the proembryo is conserved. However, we know few details of the signaling pathways for maternal tissue-to-proembryo communication.

The plant proembryo consists of an embryo-proper domain and a suspensor domain. The suspensor is necessary for embryonic development and plant fertility^8,9^ and is functionally similar to the umbilical cord of the mammalian embryo, which transports nutrients and hormones from the mother to the embryo. Thus, the suspensor is the major channel for maternal-to-proembryo communication. However, unlike the umbilical cord, which is maintained until birth and provides support throughout embryonic morphogenesis and organ differentiation, the plant suspensor degenerates at a very early stage of embryonic development. This was first discovered over a century ago; however, why and how the suspensor structure degenerates at such an early proembryonic stage is unknown. We previously confirmed that NtCYS-NtCP14 functions as a switch to control the initiation of suspensor PCD^10^, and this provides a unique opportunity and practical research model for analysis of the maternal-to-proembryo communication via the suspensor.

Here, we identify a tobacco DELLA protein, NtCRF1, and confirm that it is a direct regulator of the suspensor specific PCD-switch *NtCYS*. We show that transiently increased GA_4_ in specific maternal tissue functions as a primary signal to trigger GA downstream signaling. NtCRF1 in the suspensor responds to the maternal GA signal to promote timely suspensor PCD. Thus, we reveal a complete GA signaling cascade that mediates the cell–cell communication between maternal tissues and the proembryo, which functions as the key molecular mechanism regulating suspensor PCD.

## Results

### NtCRF1 binds to the *NtCYS* promoter

**Fig. 1:**
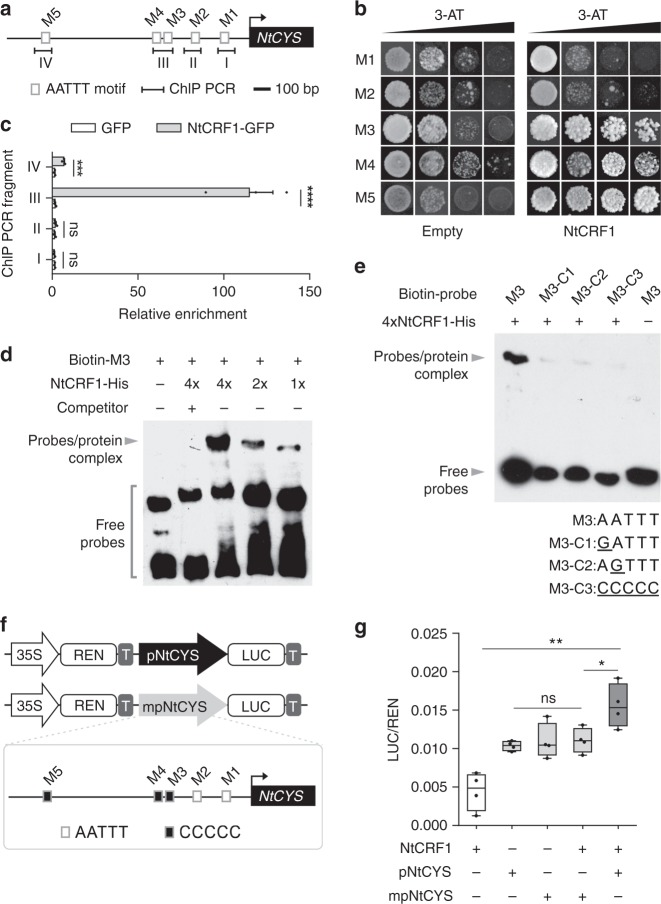
NtCRF1 binds to the *NtCYS* promoter. **a** Distribution of AATTT motifs (M) in the promoter region (−1.4 kb) of *NtCYS*. Fragments I (−189 to −83 bp), II (−396 to −296 bp), III (−609 to −476 bp), and IV (−1300 to −1211 bp) were subjected to ChIP-qPCR. **b** Yeast one-hybrid assay of the interaction of NtCRF1 with M3-M5. Empty pGAD424: control. **c** ChIP-qPCR analysis of the enrichment of NtCRF1 with the four promoter regions. *pNtCRF1::GFP*, control. Error bars represent the standard error (SE) of three biological replicates. **d** Binding of recombinant NtCRF1 to M3 was outcompeted by purified NtCRF1 (2 and 4×) and biotin-labeled DNA fragments. **e** Top: Binding of NtCRF1 to M3 mutants was reduced; Bottom: The sequences of M3 and the three mutant motifs. **f** Structure of the *pNtCYS*-driven and *mpNtCYS* (mutant promoter)-driven Dual-Luc reporter gene. 35S promoter (white arrow), *pNtCYS* (black arrow), *mpNtCYS* (gray arrow), *Renilla* luciferase (REN), firefly luciferase (LUC) and terminator (T) are indicated. Box: The mutant motifs of *mpNtCYS*. **g** Relative reporter activity (LUC/REN) in tobacco protoplasts. The relative LUC activity was normalized to the REN activity (LUC/REN, *n* = 4). (Student’s *t*-test; ns, *P* *>* 0.05; *P *<* 0.05; **P *<* 0.01; ***P *<* 0.001; ****P < 0.0001). The source data of the uncropped immunoblots are provided in the Source Data file

Based on previous work^10^, we sought to find upstream regulators of *NtCYS*. We first analyzed the promoter of *NtCYS* using PlantPan^11^ and used the transcriptome data of two-celled proembryos to select candidate transcription factor (TF)-encoding genes. We screened for candidate TFs that may interact with the *NtCYS* promoter and selected two homologs NtCRF1 (XP_009779842.1) and NtCRF2 (XP_009789076.1). These both contain GRAS domains that have been reported to bind AATTT motifs^12^. Five AATTT motifs were found in the region −1.4 kb upstream of ATG and were named M1–M5 (Fig. 1a), respectively. Using yeast one-hybrid (Y1H) assays, we confirmed that the two TFs stably interacted with the AATTT motifs in the *NtCYS* promoter (Fig. 1b and Supplementary Fig. 1a). By chromatin immunoprecipitation (ChIP)-qPCR we assayed the five AATTT motifs to verify that NtCRF1 binds to the *NtCYS* promoter, and identified the motifs that preferentially interact with NtCRF1. In the immature ovaries of a *pNtCRF1::NtCRF1-GFP* line, NtCRF1 bound to M3, M4 (*P* < 10^−4^, Student’s *t*-test), or M5 (*P* *<* 0.001, Student’s *t*-test), but not to M1 or M2 (*P* > 0.05, Student’s *t*-test) in comparison with the control line (*pNtCRF1::GFP*) (Fig. 1c). We also performed an electrophoretic mobility shift assay (EMSA) to test whether NtCRF1 directly binds to *NtCYS* promoter in vitro using the potential DNA-binding domain LHRI-VHIID-LHRII, according to a previous report^12^ (Supplementary Fig. 1b). Incubation of the *NtCYS* promoter probes, including M3–M5, with NtCRF1 LHRI-VHIID-LHRII resulted in a clear band shift (Fig. 1d and Supplementary Fig. 1c, d).

As M3 was the major binding site, we performed a competition assay using unlabeled M3 probe. The binding of NtCRF1 LHRI-VHIID-LHRII to M3 was obviously reduced by the unlabeled M3 probe, further confirming the binding between NtCRF1 LHRI-VHIID-LHRII and M3. We also mutated the AATTT motif in M3 to GATTT, AGTTT, or CCCCC (Fig. 1e); this greatly reduced the binding of NtCRF1 LHRI-VHIID-LHRII to the promoter fragments (Fig. 1e). A deletion derivative of NtCRF1 (LHRI-VHIID-LHRII) bound to M3, but not to the mutants, indicating that the LHRI-VHIID-LHRII domain is responsible for recognition of the AATTT motif (Fig. 1e). NtCRF2 yielded similar results (Supplementary Fig. 1e, f). To test whether NtCRF1 can activate expression of *NtCYS*, we utilized a dual luciferase assay based on transient transcription in protoplasts of *Nicotiana benthamiana* (Fig. 1f) and examined the effect of *NtCRF1* expression on *NtCYS* promoter activity. As shown in Fig. 1g, transiently expressed *NtCRF1* obviously promoted *NtCYS* expression in the protoplasts. In addition, we created a mutant *pNtCYS*, in which the AATTT motif in M3–M5 was replaced by CCCCC. The result confirmed that induction of *NtCYS* by NtCRF1 depended on the AATTT motif in M3–M5 (Fig. 1f, g).

**Fig. 2:**
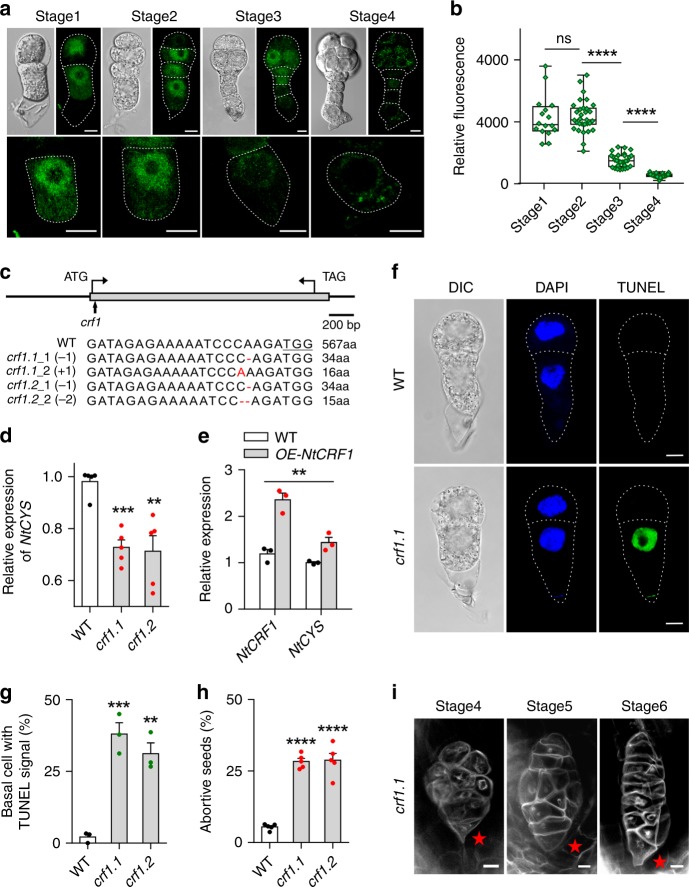
NtCRF1 promotes *NtCYS* expression and suppresses PCD in basal cells. **a** Localization of NtCRF1-GFP during early embryogenesis in *pNtCRF1::NtCRF1-GFP* plants. Magnified images of corresponding basal SCs are shown below. **b** Relative fluorescence intensity of NtCRF1-GFP in basal SCs (*n* = 16–31). **c** CRISPR/Cas9-mediated targeted mutagenesis of *NtCRF1*. Top: Schematic of *NtCRF1* showing the CRISPR/Cas9 target site (arrow). Bottom: Alignment of the sequences showing the insertion or deletion sites (red); numbers, translated amino acids (aa). **d** Expression of *NtCYS* was reduced in the *crf1* mutants. **e** Expression of *NtCYS* is increased in the *pNtCRF1::NtCRF1-GFP* line (*OE-NtCRF1*). (Two-way ANOVA, **P < 0.01). **f** Nuclear DNA fragmentation in two-celled proembryos of WT and *crf1.1*. **g** Frequency of two-celled proembryos with TUNEL-positive basal cells in WT and *crf1* (*n* = 98–109). **h** Frequency of aborted seeds in WT and *crf1* lines (*n* = 200–300 seeds per line). **i** Abnormal suspensors (stars indicated) in *crf1* lines by modified pseudo-Schiff-propidium iodide (PI) staining. Data are the means ± SE of 3 independent experiments in **e**, **g** and 5 independent experiments in **d**, **h** (Student’s *t*-test, *P < 0.05, **P < 0.01, ***P < 0.001). Scale bars: 10 μm (**a**, **f**), 20 μm (**i**). The source data of the graphs are provided in the Source Data file

To gain insight into the spatiotemporal expression pattern of *NtCRF*, we generated transgenic plants carrying the *pNtCRF1::NtCRF1-GFP* or *pNtCRF2::NtCRF2-GFP* constructs. We observed that *NtCRF1* was widely expressed in various tissues (Supplementary Fig. 2). In proembryos, NtCRF1-GFP and NtCRF2-GFP were detected in both basal and apical cell lineages, confirming expression of both NtCRF1-GFP, NtCRF2-GFP and NtCYS-GFP^10^ in the basal suspensor cell (SC) (Fig. 2a and Supplementary Fig. 3a). We also observed that NtCRF1-GFP fluorescence intensity was high in the basal cell lineage (*P* *>* 0.05 compared to stage 1, Student’s *t*-test) until stage 2, sharply declined at stage 3 (*P* < 10^−4^ compared to stage 2, Student’s *t*-test), and was scarcely detectable at stage 4 (*P* *<* 10^−4^ compared to stage 3, Student’s *t*-test) (Fig. 2b). These changes in expression coincided with those of NtCYS-GFP^10^. Therefore, NtCRF1 may positively regulate the expression of *NtCYS* in the basal SC.

### *NtCRF1* regulates *NtCYS* expression and induces suspensor PCD

To evaluate potential regulatory functions and roles in suspensor PCD, we silenced *NtCRF1* using an independent RNA interference (RNAi) construct expressed under the native promoter and selected six homozygous RNAi lines for further analysis (Supplementary Fig. 4a–c). Because *NtCRF2* mutants (Supplementary Fig. 3b) showed no seed development phenotypes (Supplementary Fig. 3c, d) and no effects on *NtCYS* expression (Supplementary Fig. 3e), we subsequently focused on *NtCRF1*. We analyzed the expression level of *NtCYS* in 5-DAP seeds (5 days after pollination; Stage 2) to assess the influence of *NtCRF1* downregulation on *NtCYS* expression. The expression level of *NtCYS* was significantly reduced in the *NtCRF1-RNAi* lines (Supplementary Fig. 4c). To exclude any off-target effects of RNAi, we generated *Ntcrf1* mutants using the CRISPR/Cas9 system (Fig. 2c). As expected, the expression level of *NtCYS* was significantly reduced in the mutants (*P* < 0.01, Student’s *t*-test) (Fig. 2d). In addition, we determined the expression levels of *NtCYS* in *NtCRF1* overexpression lines (*pNtCRF1::NtCRF1-GFP*) which had significantly greater *NtCYS* expression (*P* *<* 0.01, Two-way ANOVA) (Fig. 2e), indicating that NtCRF1 promotes the expression of *NtCYS* and is likely involved in regulating suspensor PCD.

Next, we evaluated nuclear DNA integrity in the early proembryos of wild type (WT), *NtCRF1-RNAi*, and *Ntcrf1* lines using the terminal deoxynucleotidyl transferase dUTP nick-end labeling (TUNEL) technique. In WT plants, the TUNEL signal first appeared in the suspensor at stage 4 (32-celled embryo)^10^. However, in the *NtCRF1-RNAi* and *Ntcrf1* mutant lines, the TUNEL signal was detected at the two-celled proembryo stage (Fig. 2f, g and Supplementary Fig. 4d, e). Moreover, 10–29% of embryos in *NtCYS-RNAi* seeds aborted (Supplementary Fig. 4f–h), as did about 28% of those in *Ntcrf1* mutant seeds (Fig. 2h). Notably, the suspensor of the aborted embryos showed abnormal morphology (Fig. 2i and Supplementary Fig. 4f). Due to precocious PCD, the basal cell lineage stopped dividing and degenerated. Thus, these abnormal proembryos were generally without suspensor. As an obvious phenotype, disorganized cell division was observed in all of these abnormal embryos. Pattern formation was disturbed and proembryo development was arrested at early stages. In some of the abnormal proembryos, suspensor structure could still be observed. In fact, although PCD was initiated at stage 1 in most of the proembryos, it could also occur at stage 2 or 3 in the rest of the proembryos. Thus, suspensor-like structures could be still generated in these abnormal proembryos (Supplementary Fig. 4f).

In addition, we also tested the expression of *NtCYS* by qRT-PCR in *pNtCRF1::NtCRF1-GFP* lines at stage 2 (Fig. 2e). Although the mRNA level of *NtCRF1* was increased (Supplementary Fig. 5a), NtCRF1-GFP soon disappeared at stage 4 (Fig. 2a). In this case, the expression of *NtCYS* showed no notable change at stage 4 (Supplementary Fig. 5a) and thus, the PCD initiation occurs normally as in WT (Supplementary Fig. 5b, c). These data suggest that NtCRF1 suppresses PCD by promoting *NtCYS* expression during normal suspensor development. This anti-cell-death role of *NtCRF1* is indispensable for the formation of a functional suspensor and normal embryogenesis.

### NtCRF1 functions as a DELLA protein in early embryogenesis

Sequence analysis suggests that NtCRF1 is a member of the DELLA family (Supplementary Fig. 6). Most DELLA proteins can bind to the GA receptor GIBBERELLIN-INSENSITIVE DWARF 1 (GID1) in the presence of bioactive GA^13^. After binding to GA-GID1, DELLA proteins are rapidly degraded^14^.

**Fig. 3:**
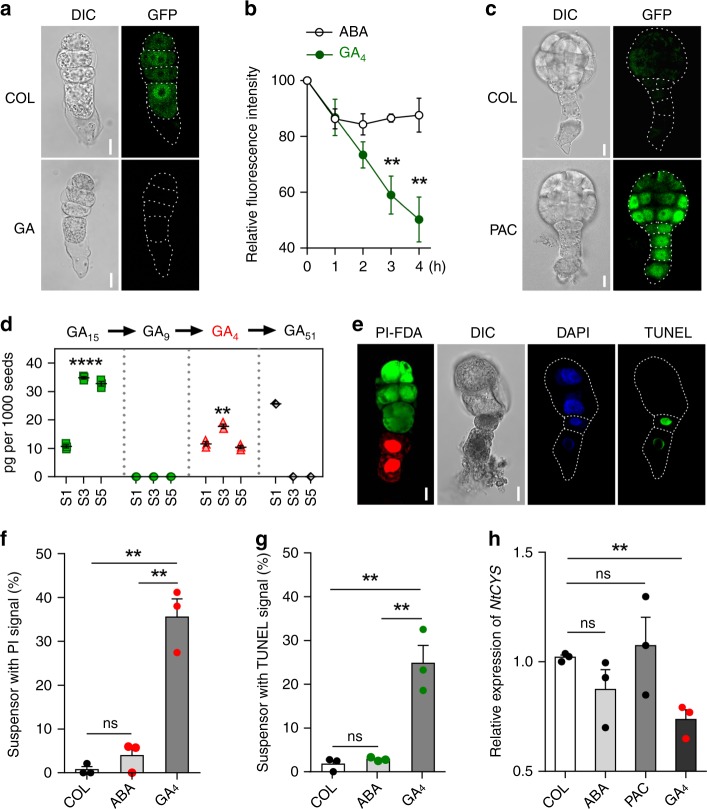
GA induces suspensor PCD via the NtCRF1-NtCYS pathway in vitro. **a** NtCRF1-GFP fluorescence in response to GA_4_ in four-celled proembryos. 0.05% ethyl alcohol as control. **b** NtCRF1-GFP fluorescence in the basal cells of two-celled proembryos during treatment with GA_4_ (or ABA) (*n* = 9). **c** NtCRF1-GFP in embryos derived from two-celled proembryo in cultured seeds after 72-h treatment with PAC. **d** Quantification of GA_15_, GA_9_, GA_4_, and GA_51_ in WT seeds at stages 1, 3, and 5 (pg per 1000 seeds, ± SE). **e** Cultured embryos stained with fluorescein diacetate (FDA), PI, and TUNEL. **f** Frequency of cultured embryos with PI-positive basal cells (*n* = 128–152). **g** Frequency of treated cultured embryos with TUNEL-positive basal cells (*n* = 106–126). **h** qRT-PCR analysis of *NtCYS* expression in 4-DAP seeds after treatment for 12-h with 100 μM ABA, PAC, or GA_4_. Data are the means ± SE of three independent experiments (Student’s *t*-test; ns, *P* *>* 0.05; **P *<* 0.01; ****P < 0.0001). Scale bars: 10 μm. The source data of the graphs are provided in the Source Data file

To determine whether NtCRF1 responds to GA, we examined NtCRF1-GFP fluorescence in the basal cell of two-celled proembryos of the *pNtCRF1::NtCRF1-GFP* line. After GA_4_ treatment, NtCRF1-GFP fluorescence was significantly reduced (Fig. 3a, b), indicating the degradation of NtCRF1. Furthermore, treatment of 4-DAP seeds of *pNtCRF1::NtCRF1-GFP* line with the GA biosynthesis inhibitor paclobutrazol (PAC) resulted in the maintenance of the NtCRF1-GFP signal until stage 5, whereas in control, the NtCRF1-GFP signal was not detected at this stage (Fig. 3c). These data suggest that NtCRF1 responds to GA and may play a role in GA signaling during suspensor PCD.

To confirm the role of NtCRF1 in GA signaling, we found all seven *NtGID1* genes in tobacco genome^15^ (Supplementary Fig. 7a). We found evidence that four were expressed in two-celled proembryos (Supplementary Fig. 7b). *NtGID1B* and *NtGID1B2* were not found in the two-celled promembryo RNA-seq dataset. Next, by yeast two-hybrid (Y2H) assay we confirmed that, in the presence of bioactive GA, NtCRF1 interacts with NtGID1B3 (XP_016477564.1), NtGID1B5 (XP_016489970.1), and NtGID1C2 (XP_016494194.1) GA receptors, respectively (Supplementary Fig. 7c). Furthermore, these three *NtGID1*s were expressed in the suspensor of a 32-celled embryo, the stage at which suspensor PCD is triggered (Supplementary Fig. 7d).

To further confirm that NtCRF1 functions as a DELLA protein, we observed seedling development in both the *NtCRF1* overexpression line L8 (*p35S::NtCRF1-GFP*) (Supplementary Fig. 8a) and *Ntcrf1.1* mutant. We found that L8 showed shorter hypocotyls (Supplementary Fig. 8b, c), whereas *Ntcrf1.1* showed longer hypocotyls. The phenotype of hypocotyl was attributed to a change in cell length, rather than cell number (Supplementary Fig. 8d-f). Furthermore, we demonstrated that these plants have altered responses to GA and PAC treatment, indicating a role of NtCRF1 in GA-induced hypocotyl growth, similar to that of known DELLAs (Supplementary Fig. 8g-j). In addition, we expressed NtCRF1-GFP and (*crf1*-Δ17)-GFP, without the DELLA domain, in tobacco BY-2 cells. We found that the GFP fluorescence in the nuclei of the BY-2 cells expressing (*crf1*-Δ17)-GFP was not affected by GA treatment, whereas nuclear fluorescence was not detectable in the BY-2 cells expressing NtCRF1-GFP after the same GA treatment (Supplementary Fig. 8k). Therefore, these data confirm that NtCRF1 functions as a DELLA protein and participates in the GA signaling pathway during early embryogenesis.

### GA as a primary signal induces suspensor PCD

Based on above results we hypothesized that GA is a primary signal that triggers suspensor PCD via NtCRF1. Therefore, we first investigated whether endogenous bioactive GA_1_ or GA_4_ are present in WT seeds at stage 1, 3 (prior to suspensor PCD), and 5 by ultra-performance liquid chromatography tandem mass spectrometry (UPLC-MS/MS). We detected GA_4_, but not GA_1_, before suspensor PCD (Fig. 3d and Supplementary Fig. 9a), suggesting that GA_4_ triggers suspensor PCD. Then, using our embryo culture system^16^, we tested whether GA_4_ is capable of inducing suspensor PCD. We cultured two-celled proembryo cells with exogenous GA_4_ or abscisic acid (ABA; control). Approximately 36% of the cultured proembryos had propidium iodide (PI)-positive and fluorescein diacetate (FDA)-negative SCs after 72-h (stage 3) (*P* *<* 0.01 compared to ABA or 0.05% ethyl alcohol, Student’s *t*-test), indicating that GA_4_ induces SC death (Fig. 3e, f). To confirm this finding, we evaluated nuclear DNA integrity in GA_4_-treated proembryos by TUNEL within 48-h (stage 2). In addition, the *NtCYS* expression was also decreased within 12-h (Fig. 3h) (*P* *<* 0.01 compared to 0.05% ethyl alcohol, Student’s *t*-test). The suspensors of 25% of proembryos were TUNEL-positive (Fig. 3e, g). We further tested the suspensor PCD when GA biosynthesis was inhibited by PAC treatment at stage 4. We found that the PCD delayed and the suspensor cells could continue dividing at the stage (Supplementary Fig. 10). Therefore, it is clear that GA_4_ as a signal induces suspensor PCD via the *NtCRF1-NtCYS* pathway.

**Fig. 4:**
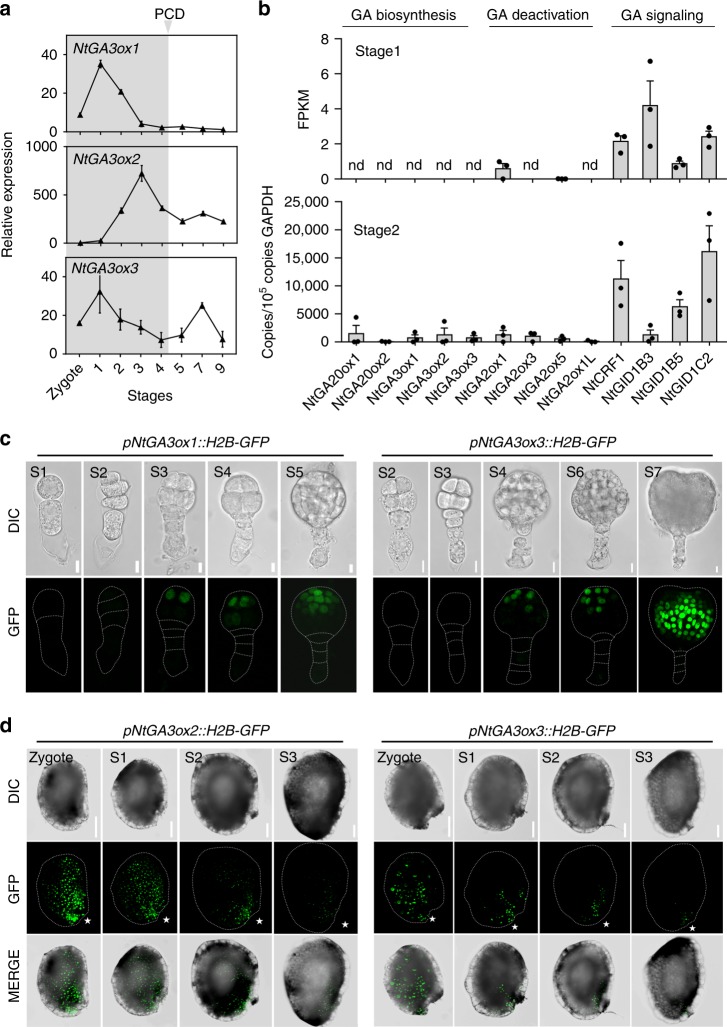
Bioactive GA biosynthesis during early embryogenesis. **a** qRT-PCR analysis of *NtGA3ox1*, *NtGA3ox2* and *NtGA3ox3* expression; *GAPDH* was used as the internal control. **b** Expression of genes related to GA biosynthesis [*NtGA20ox1* (*Ntc12*, AB012856.1) and *NtGA20ox2* (*Ntc16*, AB016084.1)], deactivation [*NtGA2ox1* (AB125232.1), *NtGA2ox1-like* (XM_016586923.1), *NtGA2ox3* (EF471117), and *NtGA2ox5* (EF471118)], and signaling in WT two-celled proembryos (stage 1) via transcriptome analysis, and via qRT-PCR analysis in WT four-celled proembryos (stage 2). *GAPDH* was used as the internal control. Values are means ± SE of three biological replicates. nd, not detected. **c** Expression of *NtGA3ox1* and *NtGA3ox3* during embryogenesis. **d** Expression of *NtGA3ox2* and *NtGA3ox3* in the developing seed coat. Asterisks, micropylar ends; Scale bars: 10 μm (**c**), 100 μm (**d**). The source data of the graphs are provided in the Source Data file

**Fig. 5:**
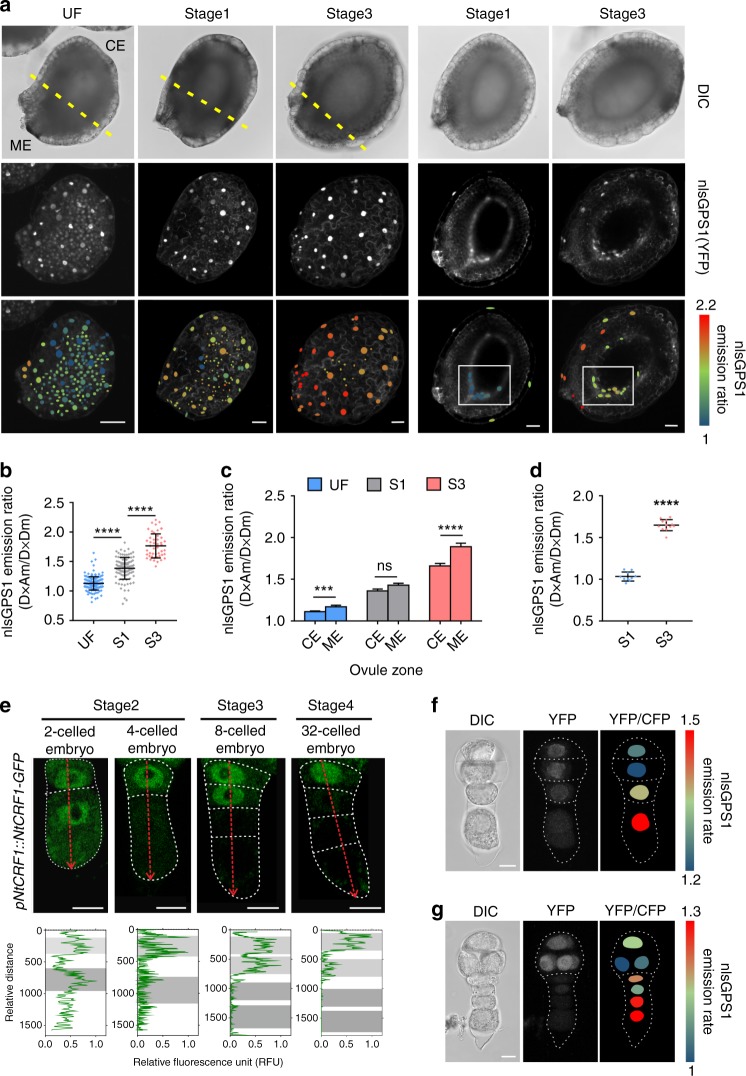
Transient increase in, and polar distribution of, bioactive GA in seeds. **a** Left: Images of the nlsGPS1 emission ratios of unfertilized (UF), 4-DAP and 6-DAP seeds. Right: Images of the nlsGPS1 emission ratios of an optical section of a 4-DAP or 6-DAP seed. Boxes: micropylar endothecium. **b** Box plot of the nlsGPS1 emission ratios of the whole seed coat (*n* > 46 nuclei). **c** nlsGPS1 emission ratios of the micropylar and chalaza ends [dashed lines in **a**; *n* > 20 nuclei]. **d** Box plot of the nlsGPS1 emission ratios of stage 1 or 3 seeds [boxed region in **a**; *n* > 10 nuclei]. **e** NtCRF1-GFP was absent from the basal SC at stage 3. Fluorescence signal intensities along a line across all SCs (red arrows). *X*-axis, relative GFP signal intensity (green); *y*-axis, relative distances along the line. Gray shading, positions of nuclei. **f** and **g** Images of the nlsGPS1 emission ratios of 4-celled and 12-celled proembryos in WT background. Data are the means ± SD (standard deviations) (Student’s *t*-test; ns, *P* > 0.05; ***P < 0.001; ****P < 0.0001). ME micropylar end, CE chalaza end. Scale bars: 100 μm (**a**), 10 μm (**e**–**g**). The source data of the graphs are provided in the Source Data file

To examine how the timing of bioactive GA accumulation may be controlled, we investigated the dynamic distribution of bioactive GA. GA3ox, a GA oxidase, catalyzes the final step of the pathway to produce GA_1_ and GA_4_. Its activity is therefore indicative of GA synthesis and distribution^17^. Thus, we cloned all homologous *GA3ox* genes expressed in seeds before suspensor PCD, including the known *NtGA3ox1* (AB032198) and *NtGA3ox2* (EF471116) genes, and a homologous *GA3ox* gene, named *NtGA3ox3* (XP_016453191.1) (Supplementary Fig. 11). We examined the expression of these *NtGA3oxs* in seeds. Interestingly, expression of the three *NtGA3ox* genes peaked prior to initiation of suspensor PCD (stage 4) (Fig. 4a) and remained at a low level thereafter. This expression pattern of key bioactive GA synthesis genes suggests a transient increase in the level of bioactive GA in seeds immediately before stage 4, which may trigger PCD of SCs. To confirm the above, we used nlsGPS1 [a nucleus-targeted variant of gibberellin perception sensor 1 (GPS1)] as a bioactive GA sensor in vivo^18^. We generated stable transgenic lines expressing *pL25::nlsGPS1. L25* was demonstrated as a reference gene with high expression stability^19^ and wide expression in vegetative tissues and seed including seed coat, endosperm, and embryo. We assessed the relative bioactive GA content by calculating the nlsGPS1 emission ratios. The results showed that the level of bioactive GA was significantly increased (*P* *<* 10^−4^, Student’s *t*-test) at stage 3 compared to that at stage 1 (Fig. 5a, b), in agreement with the *NtGA3oxs* expression (Fig. 4a) and GA_4_ content data (Fig. 3d). Thus, immediately before suspensor PCD, *NtGA3oxs* expression is upregulated, and GA_4_ is therefore transiently increased, which degrades NtCRF1 and triggers suspensor PCD.

### Maternal GA triggers suspensor PCD

We next sought to investigate the specific tissue that is the source of GA_4_ that triggers suspensor PCD. Because PCD occurs in basal SCs, we inferred that GA may be produced in the embryo, the surrounding endosperm cells, or the maternal tissue (i.e., the seed coat) and move to the basal SC. In two-celled and four-celled proembryos,  genes related to GA signaling were expressed, but those associated with GA biosynthesis and metabolism were not or at a quite low level (Fig. 4b). Next, we generated three *pNtGA3ox::H2B-GFP* constructs to determine the location of GA synthesis. *NtGA3ox1*, *NtGA3ox2*, and *NtGA3ox3* were not expressed in the endosperm and proembryo of the transgenic lines before stage 3 (Fig. 4c, d and Supplementary Fig. 9c, d). Furthermore, the three *NtGA3oxs* were not expressed in the suspensor of a 32-celled embryo, the stage at which suspensor PCD is triggered (Supplementary Fig. 9b). However, these genes were expressed in the seed coat and towards the micropyle region up to stage 4 (Fig. 4d and Supplementary Fig. 9d). This suggests that the GA_4_ that triggers PCD likely originates from maternal tissue, particularly the micropyle part of the seed coat.

Accordingly, we found that bioactive GA exhibited a polar distribution in developing seeds: the mean nlsGPS1 emission ratio increased significantly from the chalaza to the micropylar ends (*P* *<* 10^−4^, Student’s *t*-test) (Fig. 5a, c). This is in agreement with the polar activation of *NtGA3oxs* at the same stage (Fig. 4d and Supplementary Fig. 9d). Furthermore, we found that bioactive GA was localized mainly to the inner layer of the seed coat, the endothelium. The bioactive GA signal arose in the micropyle pole of the endothelial layer (Fig. 5a, d), to which the suspensor is connected. Therefore, it is clear that the bioactive GA level is transiently increased in the micropylar endothelium cells adjacent to the basal SC due to timely activation of *NtGA3oxs*.

Thus, maternal GA likely enters the basal SC and induces the GA response. To confirm this, we analyzed active GA signaling in the *pNtCRF1::NtCRF1-GFP* line. Before stage 2, we observed a strong GFP signal in the basal SC, which rapidly diminished as the GA level in the micropyle endothelium increased (Fig. 5e), indicating activation of GA signaling in the basal SC. Interestingly, the GFP signal gradually declined from the basal SC to the vertically adjacent cell (Fig. 5e), as did the occurrence of PCD. The nlsGPS1 emission ratio indicated that bioactive GA appeared in the basal SC at stages 2 and 3 (Fig. 5f, g), and subsequently moved to the vertically adjacent cell along with a decrease in the NtCRF1-GFP signal. This is in consistent with sequential decline of NtCRF1-GFP and occurrence of PCD. Therefore, entry to the basal cell of maternal GA triggers suspensor PCD.

**Fig. 6:**
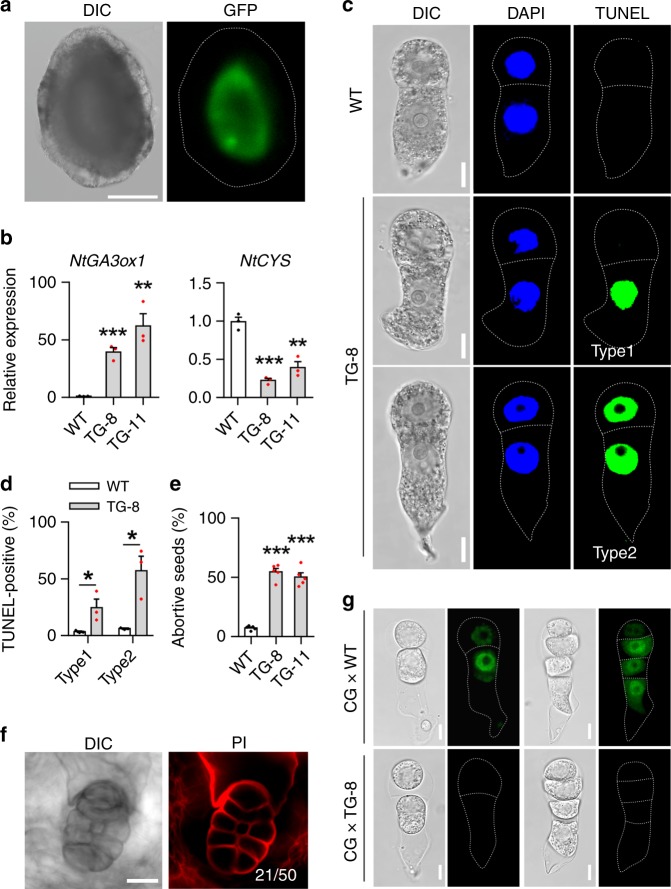
Ectopic expression of *NtGA3ox1-GFP* in the endothelium triggers PCD. **a** Localization of NtGA3ox1-GFP in the endothelium of *pNtTPE8::NtGA3ox1-GFP* plant. **b** Increased expression of *NtGA3ox1* and decreased expression of *NtCYS* in the TG-8 and TG-11 lines. **c** TUNEL test in two-celled proembryos from WT and TG-8. **d** Frequency of two-celled proembryos with TUNEL-positive apical and basal cells in WT and TG-8 (*n* = 105–112). **e** Frequency of aborted seeds in WT and TG-8. (*n* = 1000–1500). **f** PI-staining of abnormal embryos with no suspensor in the TG-8 line. **g** NtCRF1-GFP signal in early proembryos after crossing WT and *pNtCRF1::NtCRF1-GFP* (CG) lines. Data are the means ± SE of 3 independent experiments in (**b**, **c**) and 5 in **e** (Student’s *t*-test, *P < 0.05, **P < 0.01, ***P < 0.001); Scale bars: 150 μm (**a**), 10 μm (**c**, **g**), 20 μm (**f**). The source data of the graphs are provided in the Source Data file

To further confirm the induction of suspensor PCD by maternal GA in vivo, we ectopically expressed *NtGA3ox1* in the endothelium, endosperm, proembryo, and seed coat under the control of four tissue-specific promoters, respectively (Fig. 6a and Supplementary Fig. 12a). Ectopic expression of *NtGA3ox1-GFP* in the seed coat, endosperm, and proembryo did not influence *NtCYS* expression (Supplementary Fig. 12 b–d). However, ectopic expression of *NtGA3ox1-GFP* in the endothelium, to which the basal SC is connected, downregulated *NtCYS* expression (Fig. 6b). We also evaluated proembryo development in the *pTPE8::NtGA3ox1-GFP* (TG) lines. As expected, we detected PCD at the two-celled proembryo stage, as in the *NtCRF1* downregulation and *NtCYS*-*RNAi* lines. The basal cells in approximately 25% of the two-celled proembryos contained TUNEL-positive nuclei (type 1), as did both cell types of around 57% of the two-celled proembryos (type 2) (Fig. 6c, d). This basal cell PCD prevents suspensor establishment during embryogenesis (Fig. 6f), resulting in the abortion of about 50% of seeds (Fig. 6e). Next, we crossed the *pNtCRF1::NtCRF1-GFP* line with the TG-8 line to monitor the dynamics of NtCRF1 in the presence of ectopic expression of *NtGA3ox1-GFP* in the endothelium. As expected, NtCRF1-GFP fluorescence was absent from the four-celled and two-celled proembryos (13/24) (Fig. 6g). Therefore, suspensor PCD is triggered, via NtCRF1, by bioactive GA from maternal endothelial cells.

In addition, we also tried to look at the relevant aspects in Arabidopsis. As reported, *AtGA3oxs* are inactive during early embryogenesis^17^. Transcriptome analysis of *GA3oxs* and *GA20oxs* also suggests that GA biosynthesis is inactive in early proembryos^20^. GA signaling activation after fertilization specifically occurs in the seed coat of Arabidopsis^21^. These data suggest that at least the location of GA biosynthesis in maternal tissue might be conserved during early embryogenesis. Thus, we further tested the influence of loss-of-*DELLA* function or deficient GA on the suspensor development in Arabidopsis. Indeed, the *global*-*della* mutant caused defected seeds and reduced fertility (Supplementary Fig. 13a), similar to that previously reported^22^. Moreover, we found that abnormal cell division was triggered in the suspensors of these abortive seeds (Supplementary Fig. 13b), suggesting that loss-of-*DELLA* function promotes suspensor further development instead of PCD. For genetic evidence, we attempted to use WT pollen to fertilize the GA-deficient mutant *ga1-3* in Landsberg *erecta* (L*er*) background. Unfortunately, *ga1-3* mutant showed defective pistils (Supplementary Fig. 13c) and infertile ovules (Supplementary Fig. 13d), similar to that observed in *ga1-3* in Columbia background^23^, *ga1-1*^24,^ and *ga20ox* triple mutant^23^. As a result we were unable to conclusively analyze the maternal effects of GA on suspensor PCD in Arabidopsis.

## Discussion

In summary, we demonstrated that NtCRF1 binds to the M3 motif of the *NtCYS* promoter. In general, DELLA proteins do not contain classical DNA-binding domains, and thus require another TF to bind to promoter and modulate gene expression^25,26^. However, we found that NtCRF1 that has characteristics typical of DELLA proteins in response to GA can bind to the *NtCYS* promoter. Recently, a crystal structure analysis revealed that the GRAS domain of OsSCL7 binds to DNA in a dimer form^27^. We also found that NtCRF1 possesses both DELLA and GRAS domains (Supplementary Fig. 6b), and NtCRF1 and NtCRF2 could form a homo-dimer or hetero-dimer by Y2H and that the GRAS domain of NtCRF1 may mediate its binding to the promoter of *NtCYS* (Supplementary Fig. 1b, g) as AtSHR and AtSCR interact directly with the promoters^28^. Moreover, we confirmed that NtCRF1 promotes the expression of *NtCYS*, and degradation of the former at stage 3 reduces *NtCYS* expression, resulting in the activation of NtCP14 and thence suspensor PCD. Thus, maternal GA signal and proembryo responsor NtCRF1 mediate maternal-proembryo communication via the GA-GID1-CRF1-CYS-CP14 signaling cascade to precisely control the maternal-embryo connection (Supplementary Fig. 14).

It was reported that GA could trigger tapetum PCD in flowering plants via the GAMYB transcription factor^29^, which is involved in reproductive development during land plant evolution^30^. Interestingly, we also found seven *GAMYB-*like genes expressed in tobacco two-celled proembryos, and thus, it is worthy to investigate the relationship between GAMYB and suspensor PCD in the near future. In fact, during land-plant evolution, the GA-DELLA regulatory mechanism arose via independent stepwise recruitment of the GA-stimulated GID1-DELLA interaction^13^. In earlier stages [~430 million years ago (MYA)], GA was not involved in the GID1-DELLA interaction. Later, GID1-DELLA interactions became susceptible to potentiation by bioactive GA during the period of the bryophyte and lycophyte divergences (~430–400 MYA)^13^. Notably, an embryonic suspensor-like structure was first generated in low plants during the same period^8^, indicating that during plant evolution the establishment of the GA-dependent GID1-DELLA interaction was coupled to that of the suspensor structure. Thus, GA signaling is likely involved in maternal tissue-to-proembryo communication as it evolved in lower plants and maternal control of the connection between maternal tissue and the embryo might be an inborn character of embryogenesis for precise control of the duration of auxin and nutrient supply, which is critical for the embryos to establish full ability for independent development before their detachment from mother plant body.

## Methods

### Materials

Arabidopsis plants were grown in 16 h light/8 h dark conditions at 20–22 °C. *N. tabacum* L. cv. Petite Havana SR1 tobacco plants were grown under a 16/8 h light/dark cycle at 25 °C. *Ntcrf* mutants were generated using the clustered regularly interspaced short palindromic repeats (CRISPR)/CRISPR-associated protein 9 (Cas9) system^31,32^. The CRISPR target of *NtCRF* was selected as described^31,32^, and the vectors were transferred into SR1. The genomic region surrounding the CRISPR target sites of *NtCRF* were amplified by PCR, and the segment was subjected to Sanger sequencing to screen for mutants. T2 plants were used for phenotyping and T-DNA was already separated away, and identified using CRISPR/Cas9 vector-specific primers. Primer sequences are listed in Supplementary Data 1.

### Screening for *NtCYS* regulative factors

Based on our previous work on transcript profiling of two-celled proembryo, we first screened all the TFs expressed in the two-celled proembryos. We found 1267 candidates that belong to 35 gene families. Then, we analyzed the promoter sequence of *NtCYS* via PlantPan^11^. We found five AATTT motifs in *NtCYS* promoter according to the report that GRAS could bind to the AATTT motif^12^. Thus, we only focused on GRAS family that expressed in the two-celled proembryos. We isolated these five motifs embedded within a 25-bp region of the surrounding promoter sequence according to the required length of a bait for Y1H^12^.

### Yeast one-hybrid assays

Yeast one-hybrid assays were conducted using the Matchmaker One-Hybrid System (Clontech). The 25-bp AATTT region surrounding the *NtCYS* promoter sequences was synthesized with *EcoR*I and *Xba*I flanking restriction sites, and cloned into an *EcoR*I-*Xba*I-digested pHISi-1 bait vector. For expression in yeast, *NtCRF1*, *NtCRF2*, and other TF cDNAs were cloned into the pGAD424 prey vector. *Saccharomyces cerevisiae* YM4271 (Clontech) was used as the host. Transformation was confirmed by growth on SD medium containing 0, 15, 30, or 60 mM 3-AT (a competitive inhibitor of the *His3* gene product) (A8056; Sigma). The primers used are shown in Supplementary Data 1.

### Yeast two-hybrid assays

Yeast two-hybrid assays were performed according to the manufacturer’s protocol (Clontech). Briefly, *S*. *cerevisiae* strain AH109 was transformed with the bait construct pGBKT7-NtGID1s and subsequently with pGADT7-NtCRF1. Vectors lacking coding region insertions were used as negative controls. Protein interactions were scored based on growth on defined synthetic medium lacking Trp, Leu, His, and adenine in the presence or absence of 10 μM GA_1_ (G377495; TRC) and GA_4_ (G7276; Sigma). The primers used are shown in Supplementary Data 1.

### ChIP-qPCR analysis

Pooled unfertilized ovaries (~1 g) from *pNtCRF1::NtCRF1-GFP* and *pNtCRF1::GFP* plants were cross-linked under vacuum in 1% (w/v) formaldehyde for 30 min at room temperature. Cross-linking was quenched by the addition of 0.125 M glycine and vacuum infiltration continued for a further 15 min. The cross-linked tissues were frozen in liquid nitrogen and ground to a fine powder. ChIP assays were performed using the ChIP Kit-Plants (ab117137; Abcam). Nuclei were extracted and chromatin was disrupted using an Ultrasonic Cell Disruptor (JY92-IIN; Scientz) with 10 cycles on ice (15 s each at 30% duty cycle/1 min cooling). Chromatin (150 µl) was immunoprecipitated with 4 μl of an anti-GFP antibody (ab290; Abcam) for 90 min. The purified DNA fragments were used as the template for qPCR. qPCR was performed using the FastStart Essential DNA Green Master Mix (22682700; Roche) with three technical replicates, and threshold cycle numbers (Ct) were determined using a CFX Connect Real-Time PCR Detection System (Bio-Rad). Fold enrichment of the targeted genomic sequences over IgG was calculated as: 2 ^−(Ct^_CHIP_^− Ct^_IgG_^)^, where Ct_ChIP_ and Ct_IgG_ are the mean threshold cycles of triplicate PCRs of DNA samples immunoprecipitated using an anti-GFP antibody and the control IgG, respectively. All qPCR reactions were subjected to melt curve analyses and agarose gel electrophoresis to confirm the presence of a single specific product. The qPCR primer sequences are listed in Supplementary Data 1.

### Electrophoretic mobility shift assay

The DNA-interaction domain of NtCRF1 (153–396 aa) and NtCRF2 (207–417 aa) was inserted into pET32a (Novagen), respectively. The resulting plasmid was transformed into *Escherichia coli* BL21 (DE3). The recombinant NtCRF1-His protein was expressed and purified according to the manufacturer’s instructions. The 55-bp region of the promoter sequence surrounding AATTT was synthesized and the 5′-end was biotinylated (GenScript) and dissolved in a buffer (10 mM Tris and 1 mM EDTA). EMSAs were performed using the LightShift Chemiluminescent EMSA Kit (20148; Thermofisher). The labeled oligonucleotides were added to a 20-μl reaction mixture composed of 1–4 μg of the protein extract, 20 fmol biotin end-labeled target DNA, 4 pmol unlabeled target DNA (competitor), 1× DNA binding buffer, 1 μg poly(dI-dC), 2.5% glycerol, 0.05% NP-40, 50 mM KCl, and 5 mM MgCl_2_. After incubation for 20 min at 25 °C, the reaction products were resolved in 6% native polyacrylamide gels with 0.5× TBE buffer. The blots were developed using the Chemiluminescent Nucleic Acid Detection Module Kit (89880; Thermo Scientific). The primers used are shown in Supplementary Data 1.

### Transient transcription dual-luciferase assay

The reporters were constructed based on pGreenII 0800-LUC vector^33^ and the effectors were constructed based on the pGreenII 62-SK vector. *NtCYS* promoter and the mutant promoter were cloned into the *Sal*I and *Nco*I site of the pGreenII 0800-LUC vector, respectively. The full length of *NtCRF1* CDS was cloned into the *Xba*I and *Kpn*I site of the pGreenII 62-SK vector. The protoplasts were isolated from mesophyll cells of *N. benthamiana*. The plasmids were transfected into the protoplasts followed by 16 h incubation in the dark at 25 °C. The enzyme activity was measured using Dual-Luciferase® Reporter Assay System (E1910; Promega) in a microplate reader Biotek® Cytation3. Four independent measurements were carried out for the analysis.

### Quantification of endogenous GAs

For determination of endogenous gibberellin in the seeds at stage −1, −3, and −5, we collected and grinded 500 mg seeds into powder as one sample after quick freezing with liquid nitrogen. Each sample was extracted with 5 ml of 90% aqueous methanol (MeOH) and 2 ng of each D-labeled GA compound as internal standards. The MAX cartridge (Waters) was activated and equilibrated with 10 ml MeOH, water, 5% NH_4_OH, and 90% MeOH in turn, while MCX (Waters) with 10 ml MeOH, water and 90% MeOH in order. Then the MAX cartridge was disconnected and washed with 5% NH_4_OH in 5% MeOH, MeOH successively. Finally, GAs were eluted with 2% formic acid in 90% MeOH. After dried with N_2_ stream, the eluent was reconstructed with 200 μl 80% MeOH and subjected to UPLC-MS/MS analysis. GAs analysis was performed on a quadrupole linear ion trap hybrid mass spectrometer (QTRAP 5500, AB SCIEX) equipped with an electrospray ionization source coupled with a UPLC (Waters). Five microliter of each sample was injected into a BEH C18 column. GAs were detected in negative multiple reaction monitoring (MRM) mode. Each GA compound was quantified with a MRM transition and qualified with another one. Three independent samples were analyzed for each stage.

### Identification of *NtGA3ox* genes

We firstly found *GA3ox3* according the reference Ntab-TN90 Primary Assembly (BioProject: PRJNA319578) and its GO (Gene Ontology) annotation is ‘gibberellin 3-beta-dioxygenase 4-like’. Except two known *GA3oxs*, we found five annotated *GA3ox*-like genes in *N. tabacum* (Supplementary Fig. 11a). By sequence analysis, we chose the four of them for further analysis since the sequence of another one is distinctive compared to GA3oxs (Supplementary Fig. 11b). We tested the six *GA3oxs* and found three of them (*NtGA3ox*-1, -2, and -3) expressed in seeds before suspensor PCD. Thus, *NtGA3ox*-1, -2, and -3 were used for further analysis.

### Embryo isolation and seed clearing

For embryo isolation, seeds were first treated in enzyme solution (1% cellulase R10, 0.5% macerozyme R10, 10.5% mannitol, and 3 mM MES, pH 5.5) for 30 min in the dark. Then droplets of the seed suspension were gently grinded by a flat-headed glass rod. After grinding several droplets of 10.5% mannitol were added for releasing and washing embryo sacs. Living embryos could be further isolated by a second enzymatic maceration procedure followed by a brief micromanipulation.

For seed clearing, whole seeds were collected in a 2ml-centrifuge tube and were fixed by 50% methanol and 10% acetic acid at 4 °C for 12-h and then subjected to an overnight treatment of 1% SDS and 0.2 M NaOH at room temperature. Thereafter, the seeds were rinsed in water, followed by an incubation in 2.5% NaClO for 1-h, and then rinsed again. Next, the seeds were transferred into 1% periodic acid for 1-h at room temperature and in 80% ethanol for 10 min at 80 °C. Subsequently, the seeds were transferred back to the same fixative and incubated for 1-h before the seeds were rinsed again with water. After washing, the seeds were incubated in Schiff reagent with propidium iodide (PI) (P4170; Sigma) (100 mM sodium metabisulphite and 0.15 M HCl; PI to a final concentration of 0.1 mg/ml) for about 3-h. The samples were stored in a chloral hydrate solution (4 g chloral hydrate, 1 ml glycerol, and 2 ml water) and kept overnight at room temperature. Next, the chloral hydrate was replaced by Hoyer’s solution (30 g gum arabic, 200 g chloral hydrate, 20 g glycerol, and 50 ml water). The seeds were kept in the Hoyer’s solution for at least 10 days before observation under a confocal microscope.

### RNA-seq and data analysis

mRNA of isolated two-celled proembryos (40–50 embryos per sample) was directly extracted using Dynabeads mRNA DIRECT^TM^ Micro Kit (Life Technology). cDNA preparation was performed using the SMARTer Ultra Low Input RNA Kit for Sequencing v3 (Clontech). Libraries were constructed using TruePrep^TM^ DNA Library Prep Kit V2 for Illumina (Vazyme) and then sequenced on Illumina HiSeq^TM^ 4000. Adapters and low-quality reads were filtered using Cutadapt and an in-house script. Next, clean reads were mapped to the tobacco genome (*N. tabacum* genome reference, Ntab-TN90) using Bowtie 2. Gene expression levels were quantified as FPKM (fragments per kilobase of transcript per million mapped reads) using RSEM. The raw data for each sample are available in the NCBI Gene Expression Omnibus under accession number GSE133373.

### Cell viability and DNA fragmentation analyses

To evaluate cell viability and plasma membrane integrity, we stained embryos with fluorescein diacetate (FDA) (F7378; Sigma) and PI. Isolated embryos were incubated in the solution containing 10.5% mannitol, 2 µg/ml FDA, and 1 µg/ml PI for 15 min at room temperature and washed twice with 10.5% mannitol before observation. To detect nuclear DNA fragmentation, the isolated embryos were tested by DeadEnd^TM^ Fluorometric TUNEL System (G3250; Promega) or In Situ Cell Death Detection Kit, TMR red (12156792910; Roche) for *pNtCRF1::NtCRF1-GFP* line according to the manufacturer’s instructions, respectively.

### Two-celled proembryo culture in vitro

The ovaries were rapidly surface-sterilized with 75% alcohol, followed by 5-min washes in sterile water for three times. Seeds were then isolated from the ovaries and collected in 2 ml 13% mannitol for co-culture. The isolated two-celled proembryos were transferred into a Millicell (PICM01250; Millipore) containing 100 μl of the medium^16^. The Millicell was put in a 3-cm Petri dish containing 1.5 ml of the medium^16^, and 100–150 seeds were co-cultured as feeder at 25 °C in the dark. 1 μM GA_4_ or 1 μM ABA (A1049; Sigma) were added to the medium as appropriate.

### PAC, GA, ABA, and ethyl alcohol treatment assays

Two-celled or four-celled proembryo isolated form *pNtCRF1::NtCRF1-GFP* line and treatment with 100 μM GA_4_ for hours, 0.05% ethyl alcohol as control. Embryos derived from in cultured seeds form *pNtCRF1::NtCRF1-GFP* line after 72-h treatment with 100 μM PAC, 0.05% ethyl alcohol as control. 4-DAP WT seeds treated with 100 μM ABA, PAC (46046; Sigma), or GA_4_ for 12-h, 0.05% ethyl alcohol as control, then collected for qRT-PCR.

### Quantitative real-time PCR

Total RNA was extracted from leaves, roots, stems, anthers, sepals, and seeds using the MiniBEST Plant RNA Extraction Kit (9769; Takara). Total RNAs were treated with RNase-free DNase I (Promega), and cDNA was synthesized using Transcriptor Reverse Transcriptase (Invitrogen) according to the manufacturer’s instructions. 0.2 μl of resulting first-strand cDNA was used as a PCR template for qRT-PCR. Quantitative PCR analysis were performed using FastStart Universal SYBR Green Master (4913850001; Roche) with a CFX Connect^TM^ Real-Time System (Bio-Rad). *Glyceraldehyde-3-phosphate dehydrogenase* (*GAPDH*), or both *GAPDH* and *Ubiquitin-conjugating enzyme2* (*UBI*) were used as the internal reference(s) to normalize the relative level of each transcript. Each experiment was repeated three or five times and each time the experiment included duplicate samples. The qPCR primer sequences are listed in Supplementary Data 1. The raw data are shown in Source Data file.

### Transformation of BY-2 cells

About 4 ml of 1-week-old tobacco BY-2 culture was first transferred into a 60-mm × 15-mm Petri dish, and then 20 μl prepared *Agrobacterium tumefaciens* line GV3101, containing *p35S::NtCRF1-GFP* or *p35S::(crf1-Δ17)-GFP*, was added into the culture dishes, respectively. Then each plate was sealed with Parafilm and incubated for 3-day at 28 °C in the dark on a shaker at 100 r.p.m. Subsequently, the cultured cells were washed four times and dipped in NT1 medium^34^ containing 250 mg/L carbenicillin for 30 min to kill Agrobacterium, and then plated onto solid NT1 medium containing 250 mg/L carbenicillin and 50 mg/L kalamycin. After about 30-day, kalamycin-resistant calli appeared on the medium, and subsequently they were transferred onto new plates and suspended again in 50 ml liquid NT1 medium for observation. The cells were treated with 100 μM GA_3_ for 4-h and 0.05% ethyl alcohol as control. The primers used are shown in Supplementary Data 1.

### Confocal microscopy and image analysis

Stained embryos were observed under a confocal microscope (Leica SP8). The nlsGPS1 emission ratio was determined as described^18^. Images were processed using ImageJ or Adobe Photoshop CC.

### Quantification and statistical analysis

The fluorescence intensity was calculated as: integrated density—(area of selected cell nucleus × mean background fluorescence). qRT-PCR and fluorescence intensity data were assumed to follow normal distributions and were subjected to Student’s *t*-test or analysis of variance (ANOVA) using GraphPad Prism 7 software. The value of *n*, the definition of the center, dispersion, precision, and statistical significance are provided in the figure legends. *P *<* 0.05; **P *<* 0.01; ***P *<* 0.001; ****P *<* 0.0001 compared to mock controls, unless otherwise specified by connecting lines.

### Reporting summary

Further information on research design is available in the Nature Research Reporting Summary linked to this article.

## Supplementary information

Supplementary Information

Description of Additional Supplementary Files

Supplementary Data 1

Reporting Summary

Source Data

## Data Availability

Data supporting the findings of this study are available within the main text and its Supplementary Information files. The source data underlying Figs. 1c-e, 1g, 2b, 2d, 2e, 2g, 2h, 3b, 3d, 3f-h, 4a, 4b, 5b-d, 5f, 5g, 6b, 6d, 6e, and Supplementary Figs. 1c-f, 2a, 3d, 3e, 4c, 4e, 4g, 4h, 5a, 5c, 8c, 8e, 8f, 8g, 8i, 10b, 12b–d are provided as a Source Data file. RNA-seq data has been deposited at the NCBI Gene Expression Omnibus database under accession number GSE133373. All data are available from the corresponding author upon request.
