# Supplementary material for: Maternal control of suspensor programmed cell death via gibberellin signaling
Source: Nat Commun. 2019 Aug 2;10:3484. doi: 10.1038/s41467-019-11476-3 (PMC6677759; doi:10.1038/s41467-019-11476-3)
Supplement: Supplementary file 1 — Supplementary Information [file 41467_2019_11476_MOESM1_ESM.pdf]

Supplementary Information

Maternal control of suspensor programmed cell death via gibberellin signaling

Shi et al.

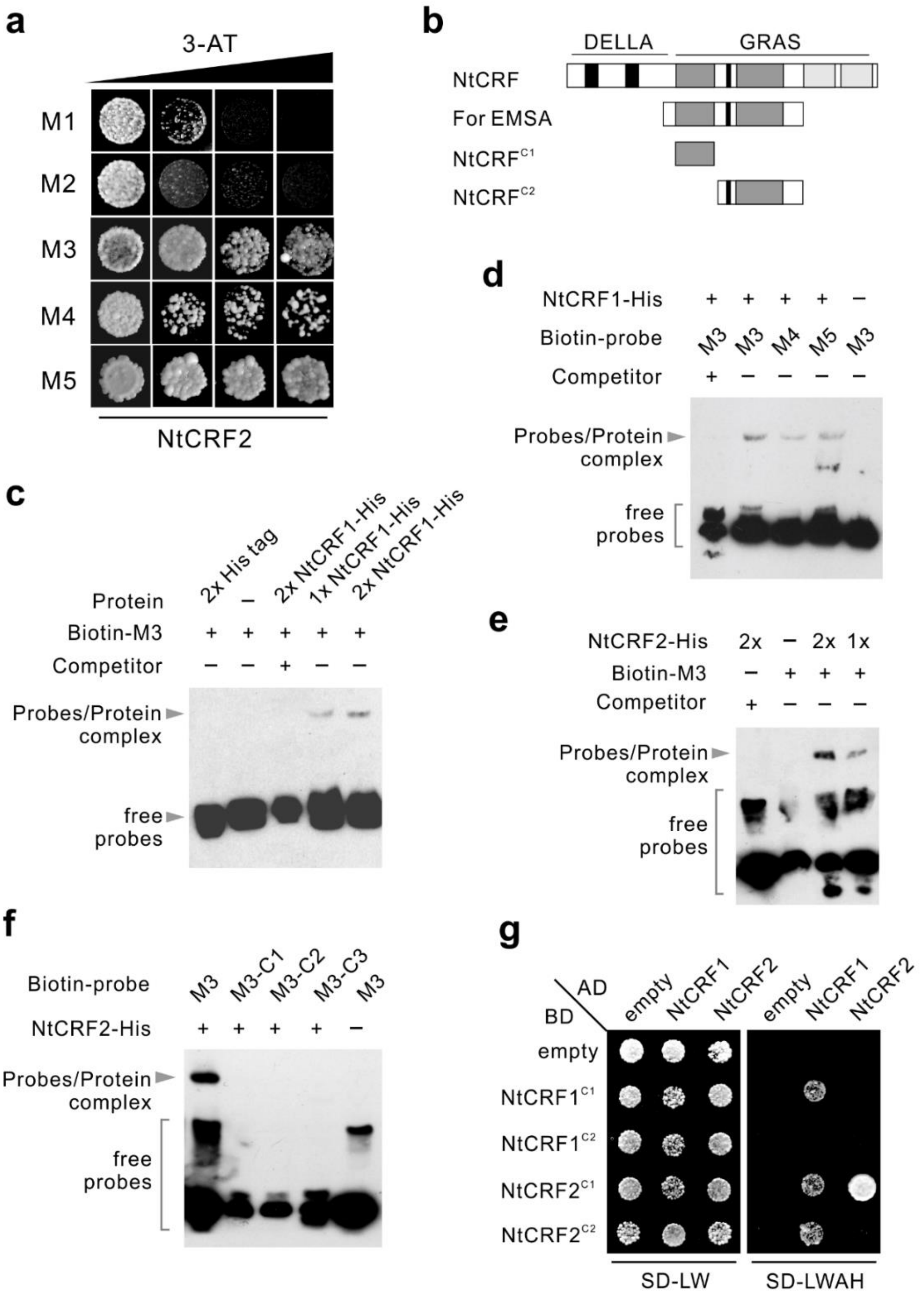

Supplementary Fig. 1: Biochemical characters of NtCRF.

a The interaction of NtCRF2 with three AATTT motifs (M1-M5) of the *NtCYS* promoter tested by yeast one-hybrid assay. **b** A schematic diagram showing the structure of NtCRF. **c** EMSA of His-tag with NtCRF1 used as a negative control. **d** EMSA of NtCRF1 with M3, M4 and M5, respectively. **e** and **f** EMSA of NtCRF2 with M3 motif of the *NtCYS* promoter. **g** NtCRF1 and NtCRF2 could form homo- or hetero-dimer by yeast two-hybrid test [domains for test is marked in (**b**)]. The source data of the uncropped immunoblots are provided in the Source Data file.

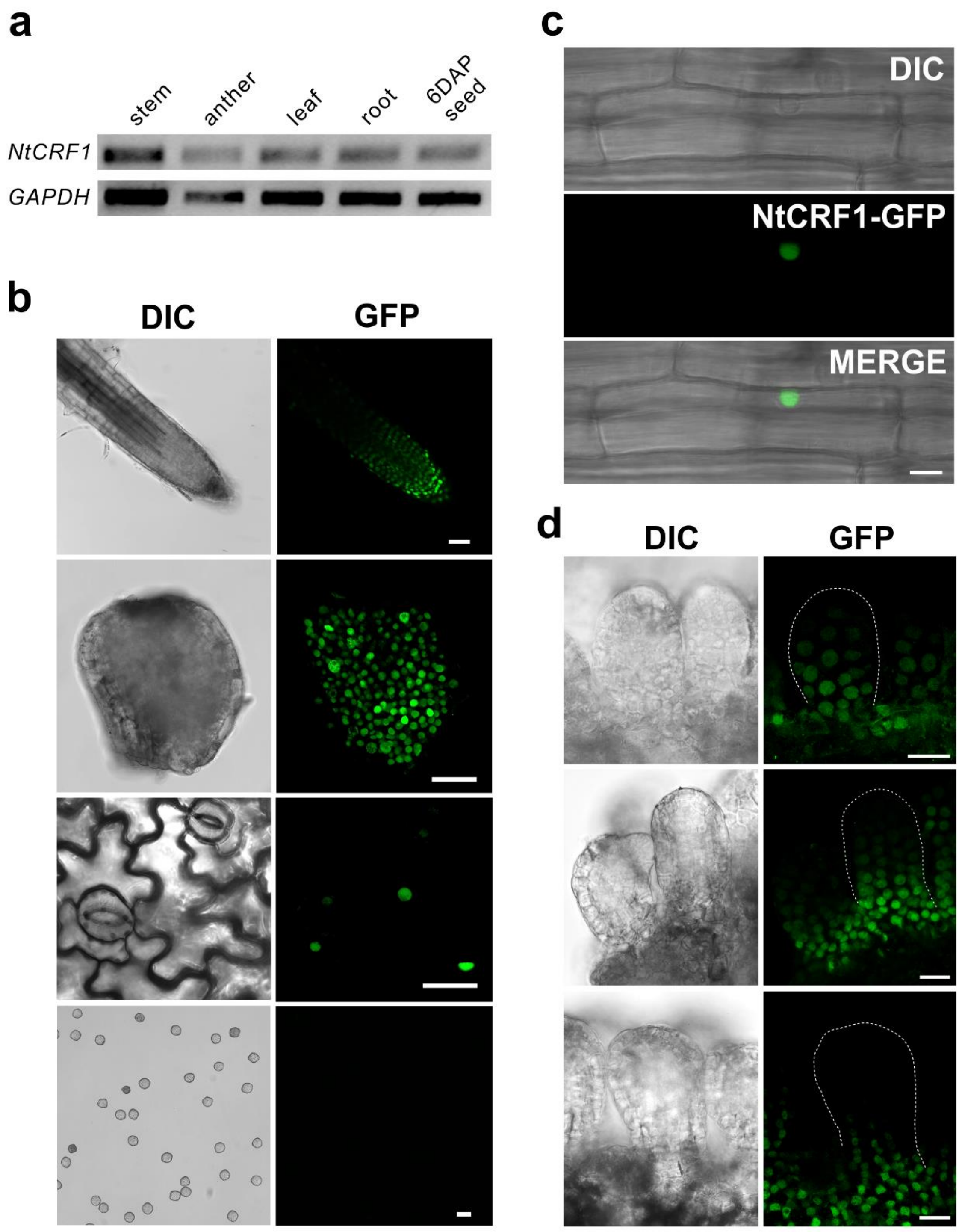

Supplementary Fig. 2: The expression pattern of *NtCRF1*.

a Semi-quantitative PCR analysis of *NtCRF1* expression in stem, anther, leaf, root and 6-DAP seeds. *GAPDH* was used as a control. **b** Expression of *pNtCRF1::H2B-GFP* in root, ovule, leaf and pollen, respectively. **c** Location of NtCRF1-GFP in the hypocotyl of *pNtCRF1::NtCRF1-GFP* line. **d** Expression of NtCRF1-GFP during ovule development in *pNtCRF1::NtCRF1-GFP* plant. Scale bars: 100 μm (**b**), 20 μm (**c**) and 50 μm (**d**). The source data of the uncropped gels are provided in the Source Data file.

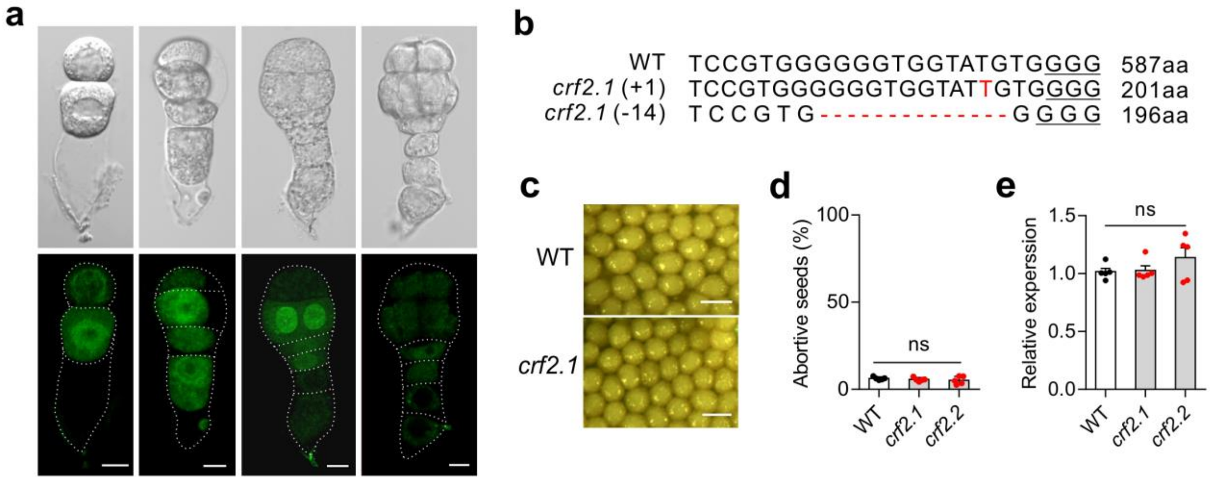

Supplementary Fig. 3: *Ntcrf2* mutants have no effects on *NtCYS* expression.

a Localization of NtCRF2-GFP during early embryogenesis in *pNtCRF2::NtCRF2-GFP* plants. Scale bars: 10μm. **b** Alignment of the sequences of WT and *crf2* mutants showing the insertion or deletion sites (red); numbers, translated amino acids (aa). **c** aborted seeds in WT and *crf2* mutant lines. Scale bars: 500μm. **d** The frequency of aborted seeds in WT and *crf2* mutant lines. Data represent the mean ± SE from five independent experiments, with 200 - 300 seeds per line in each experiment. **e** The relative expression of *NtCYS* in *crf2* mutants measured by RT-qPCR. The expression level of *NtCYS* in the WT is set to 1. Data represent the mean ± SE from three independent experiments (ns, $P > 0.05$, Sudeten's t-test). The source data of the graphs are provided in the Source Data file.

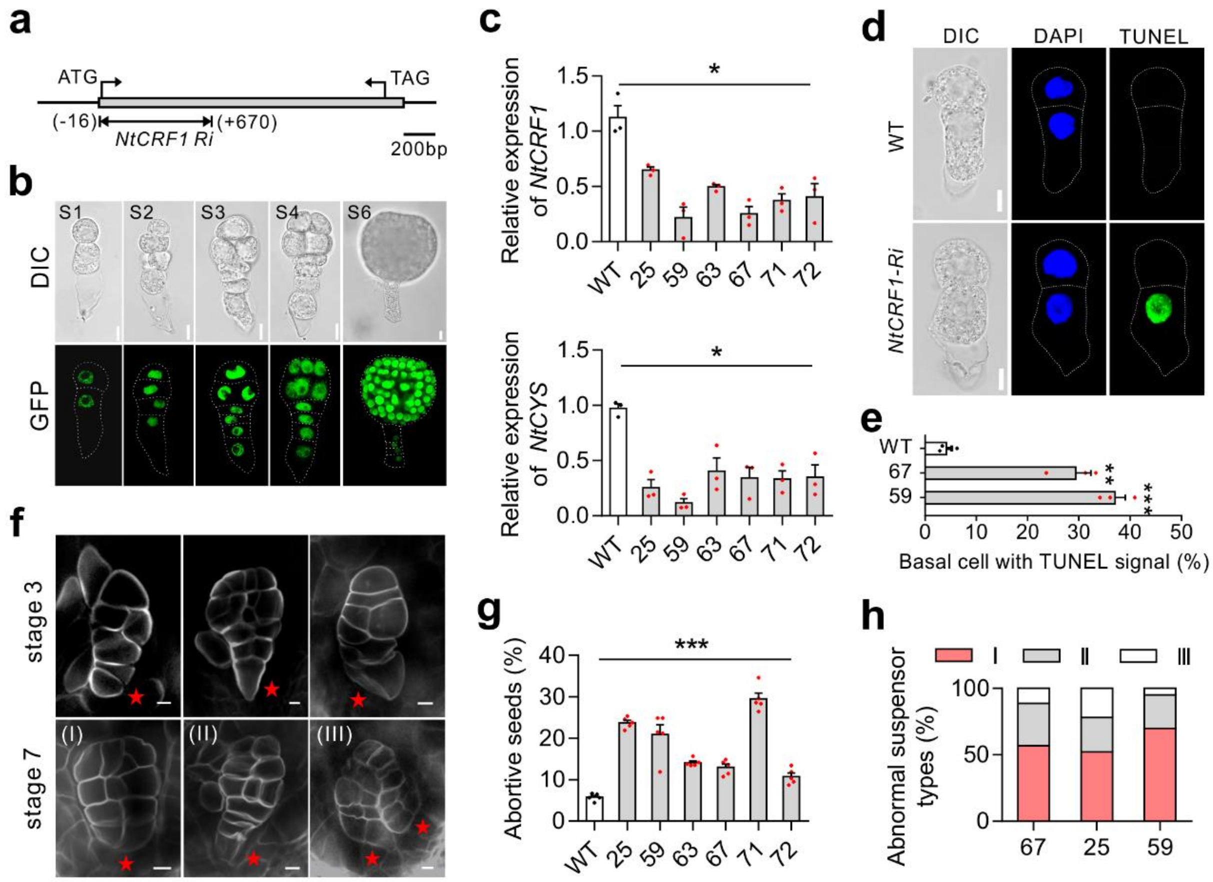

Supplementary Fig. 4: Downregulation of *NtCRF1* deceases the *NtCYS* expression and induces precocious PCD in the basal cell lineage.

a A schematic diagram of *NtCRF1* gene showing the RNAi target region. **b** Promoter activity of *NtCRF1* (*pNtCRF1::H2B-GFP*) during embryogenesis. **c** Quantitative analysis of *NtCRF1* expression in 5DAP seeds of independent *NtCRF1* RNAi (Ri) lines. Quantitative analysis of *NtCYS* expression in 5DAP seeds of corresponding *NtCRF1-Ri* lines. The levels of gene expression are normalized to *GAPDH* and *UBI* expression. The level of *NtCYS* expression in wild-type anthers is set to 1. **d** Nuclear DNA fragmentation in two-celled proembryos of WT and *NtCRF1-Ri* lines stained with TUNEL. **e**, The frequency of two-celled proembryos with TUNEL-positive basal cells in WT and *NtCRF1 Ri* lines (line 59 and 67) (n = 97 - 124). **f** The abnormal suspensors at early stages of *NtCRF1* Ri embryo showed by modified pseudo-Schiff-propidium iodide staining. Stars indicated suspensor. **g** The frequency of aborted seeds in WT and *NtCRF1-Ri* lines. (n=1,000-1,500) **h** The relative frequency of abnormal or absent suspensors in (**f**) at stage 7 (n=150). Data are the means ± SE of 3 independent experiments in (**c**, **e**) and 5 in (**g**); Scale bars: 10 µm. (Student's t-test, ns, $P > 0.05$, $^{*}P < 0.05$, $^{**}P < 0.01$, $^{***}P < 0.001$, and $^{****}P < 10^{-4}$). The source data of the graphs are provided in the Source Data file.

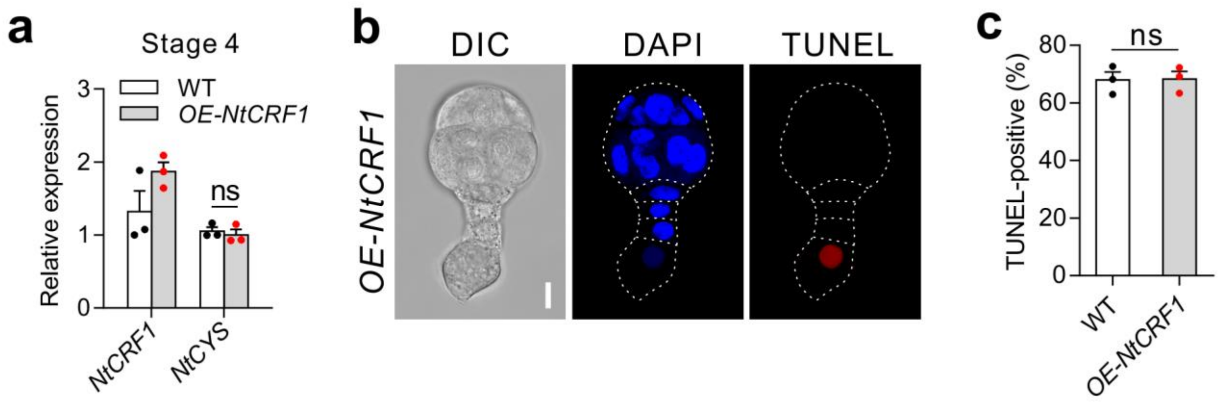

Supplementary Fig. 5: Overexpressing *NtCRF1-GFP* has no effect on suspensor PCD at stage 4.

a Relative expression of *NtCRF1* and *NtCYS* at stage 4, as measured by qRT-PCR. The expression level in WT was set to 1. **b** The Nuclear DNA fragmentation in the suspensor SC of *OE-NtCRF1* at stage 4 stained with TUNEL. **c** The frequency of embryos with TUNEL-positive basal cells in *OE-NtCRF1* line at stage 4. Data represent the mean ± SE from 3 independent experiments (n = 90) (Student's t-test, ns, $P > 0.05$). Scale bar: 10 µm. The source data of the graphs are provided in the Source Data file.

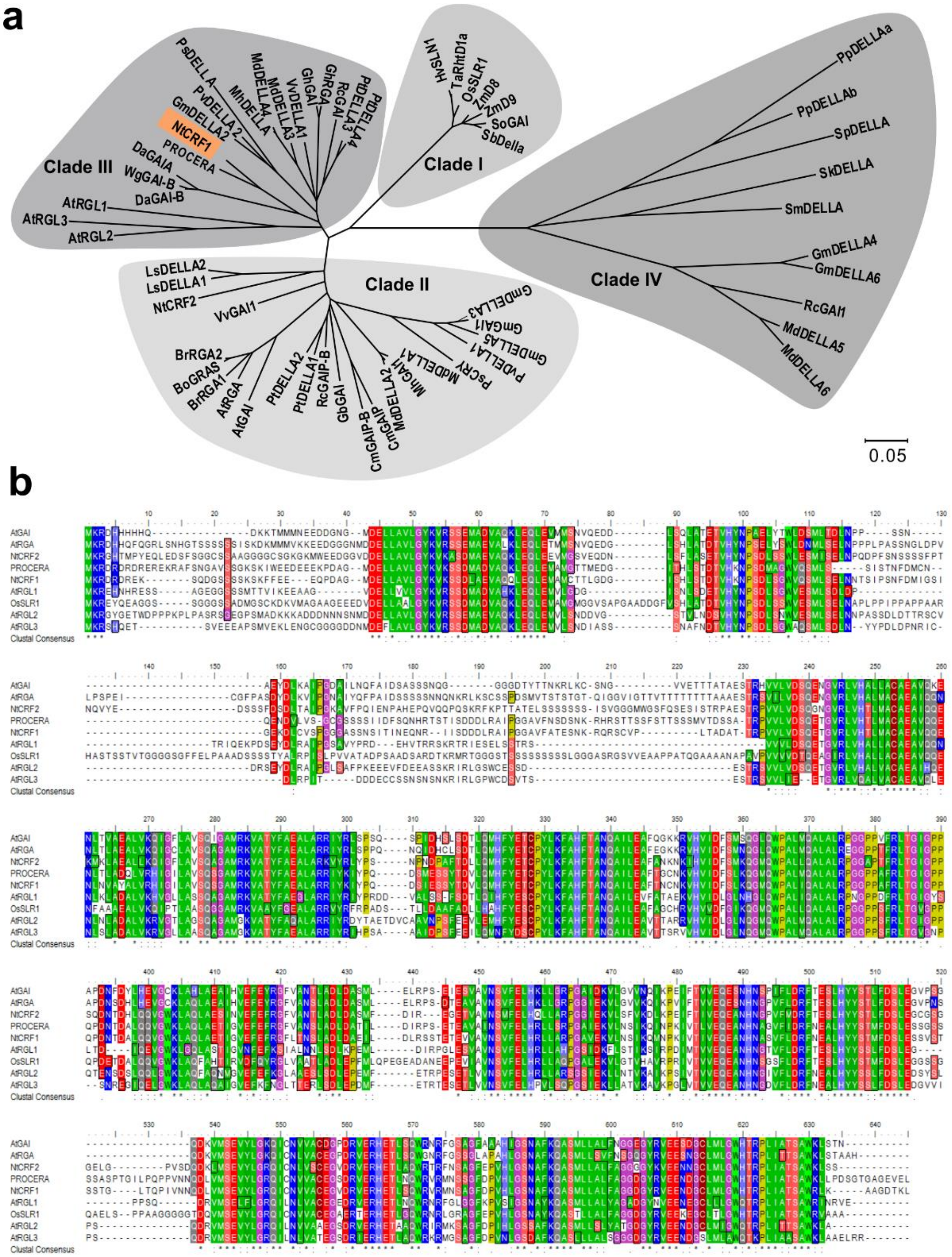

Supplementary Fig. 6: Phylogenetic analysis and sequence alignment and of NtCRF and DELLAs.

a Phylogenetic analysis of NtCRFs and DELLA homologs in plants. The scale bar indicates the number of amino acid substitutions per site. **b** Alignment of NtCRFs with the known DELLAs, including tomato DELLA (PROCERA), rice DELLA (OsSLR1) and Arabidopsis (AtRGA, AtGAI and AtRGL1, AtRGL2, AtRGL3).

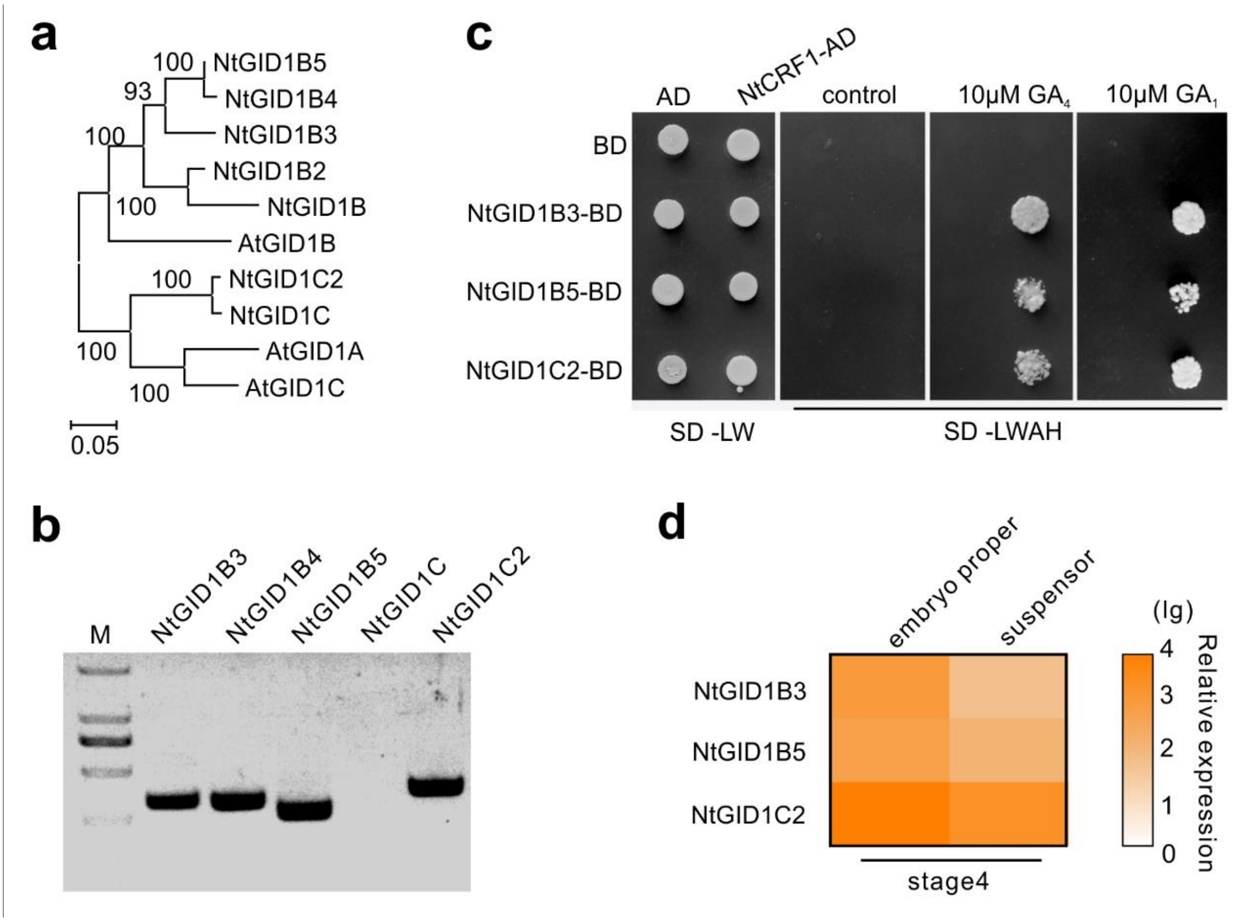

Supplementary Fig. 7: NtCRF1 interacts with NtGID1s depending on bioactive GA.

a Phylogenetic analysis of NtGID1s [NtGID1B (XP_016477389.1), NtGID1B2 (XP_016514746.1), NtGID1B4 (XP_016476806.1), and NtGID1C (XP_016452257.1)] and its three homologs in Arabidopsis. The scale bar indicates the number of amino acid substitutions per site. **b** Semi-quantitative analysis of *NtGID1* in two-celled proembryo (stage 1). **c** NtCRF1 interacts with NtGID1B3, NtGID1B5 and NtGID1C2 depending on GA_1 (10μM) or GA_4 (10μM), confirmed by yeast two-hybrid, respectively. The control is 0.01% ethyl alcohol. **d** qRT-PCR analysis of *NtGID1B3*, *NtGID1B5* and *NtGID1C2* in embryo proper and suspensor of 32-celled embryos (stage 4). The *GAPDH* was used as an internal control.

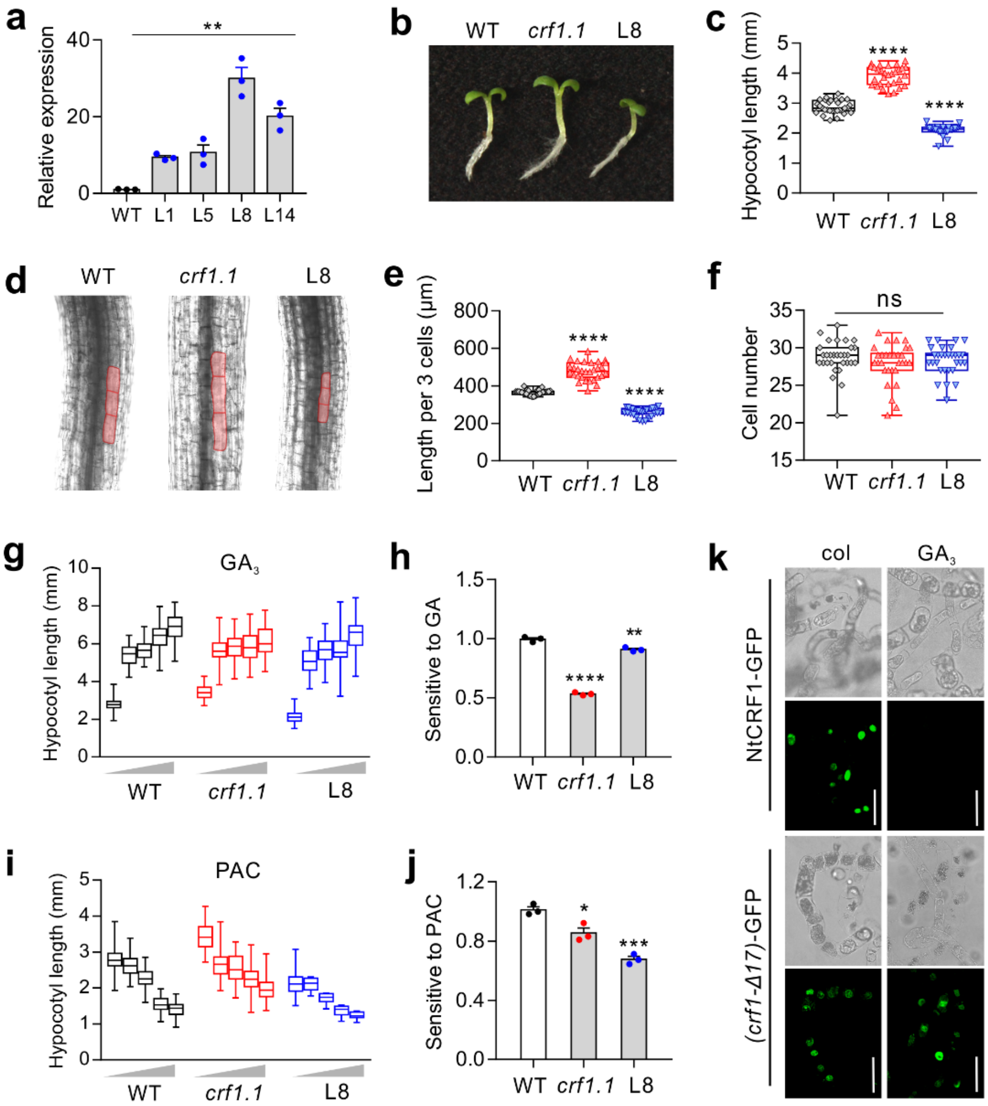

Supplementary Fig. 8: NtCRF1 shows the properties of known DELLAs.

a Relative expression level of *p35S::NtCRF1-GFP* lines. Values are means ± SE of three biological replicates. **b** and **c** Representative images of hypocotyl and the lengths of 6-d-old WT, *Ntcrf1.1* and *p35S::NtCRF1-GFP* (L8) seedlings (n = 90). **d** and **e** The lengths of 3 cells in the hypocotyl (n = 90). The sizes of the 3 cells are indicated by the red regions. **f** The cell number of the hypocotyl (n = 90). **g** Hypocotyl lengths of the seedlings grown under increasing concentrations of GA_3 (0, 0.5, 1, 2 and 5 μM). **h** The relative response to GA treatment. **i** Hypocotyl lengths of the seedlings grown in the presence of increasing concentrations of the GA biosynthesis inhibitor PAC (0, 0.1, 0.2, 0.4 and 1 μM). **j** The relative response to PAC treatment. **k** Effect of GA_3 treatment on fluorescence of transgenic BY-2 cells expressing NtCRF1-GFP and (*crf1*-Δ17)-GFP fusion protein, respectively. 0.05% ethyl alcohol was used as control. Scale bar: 100 μm. (Student's t-test, ns, $P > 0.05$, *$P < 0.05$, **$P < 0.01$, ***$P < 0.001$, and ****$P < 10^{-4}$). The source data of the graphs are provided in the Source Data file.

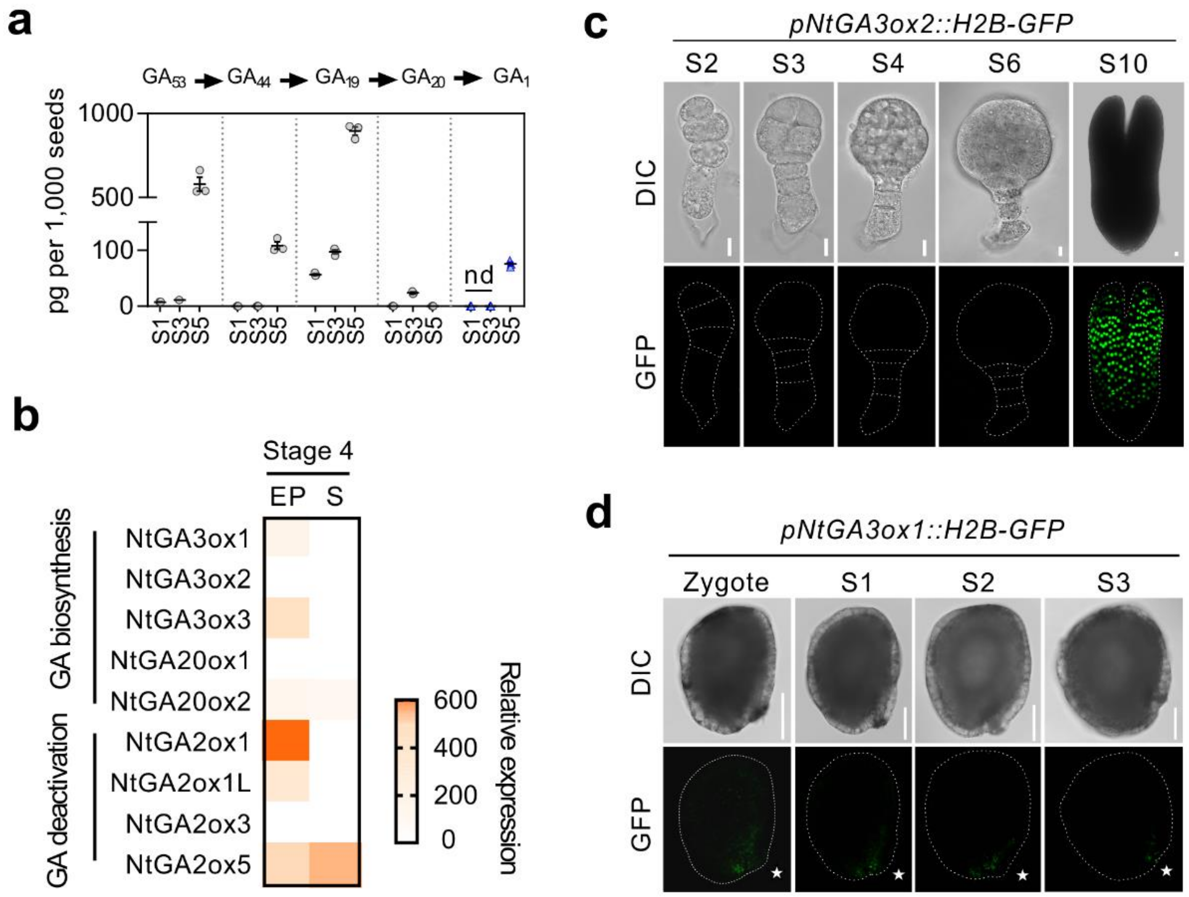

Supplementary Fig. 9: Expression of the genes related to GA biosynthesis and deactivation during embryogenesis.

a Quantification of GA_{53}, GA_{44}, GA_{19}, GA_{20} and GA_1 in WT seeds at stage 1, stage 3 and stage 5, respectively. Plotted data are the means of three replicates and presented as pg per 1,000 seeds, ± SE. nd, not detected. **b** RT-PCR analysis of GA biosynthesis and deactivation gene in embryo proper and suspensor of 32-celled embryo (stage 4). The *GAPDH* was used as an internal control. **c** Expression of *NtGA3ox2* during embryogenesis. **d** Expression of *NtGA3ox1* in the developing seed coat. Asterisks, micropylar ends; Scale bars: 10 µm (**c**), 100 µm (**d**). The source data of the graphs are provided in the Source Data file.

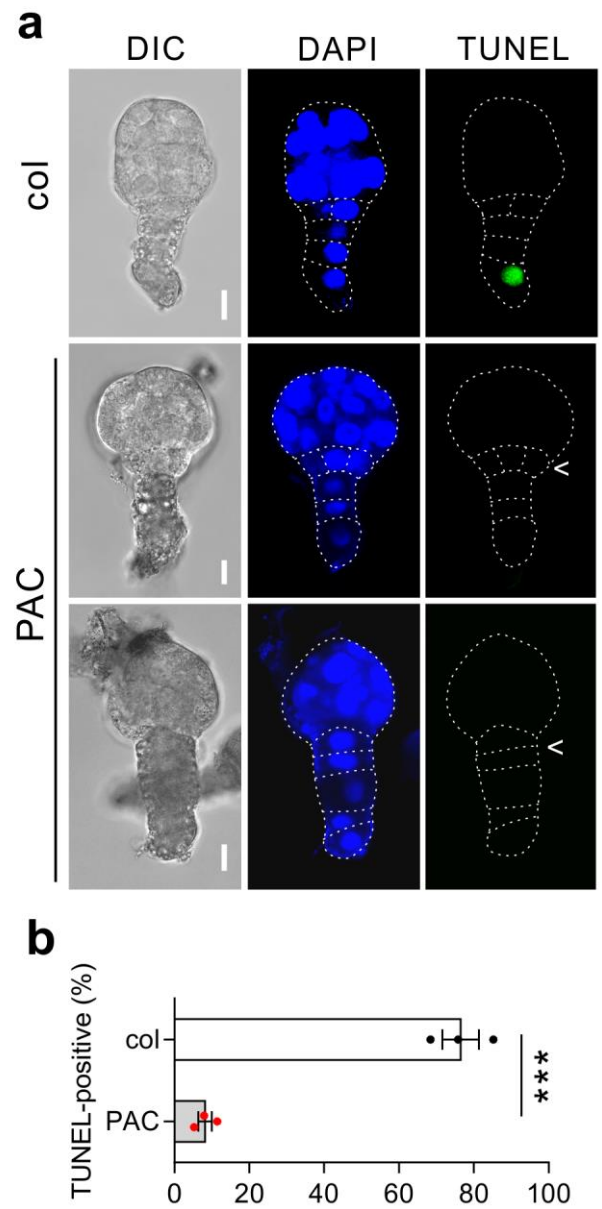

Supplementary Fig. 10: Inhibition of GA biosynthesis delayed suspensor PCD and induced extra suspensor division.

a The Nuclear DNA fragmentation in embryo at stage 4 with PAC or control (0.05% ethyl alcohol) treatment. Arrows indicate extra divisions. Scale bars: 10 µm. **b** The frequency of the embryos with TUNEL-positive basal cells at stage 4 after PAC or control treatment. Data represent the mean ± SE from 3 independent experiments (n = 105 - 120) (Student's t-test, ****P* < 0.001). The source data of the graphs are provided in the Source Data file.

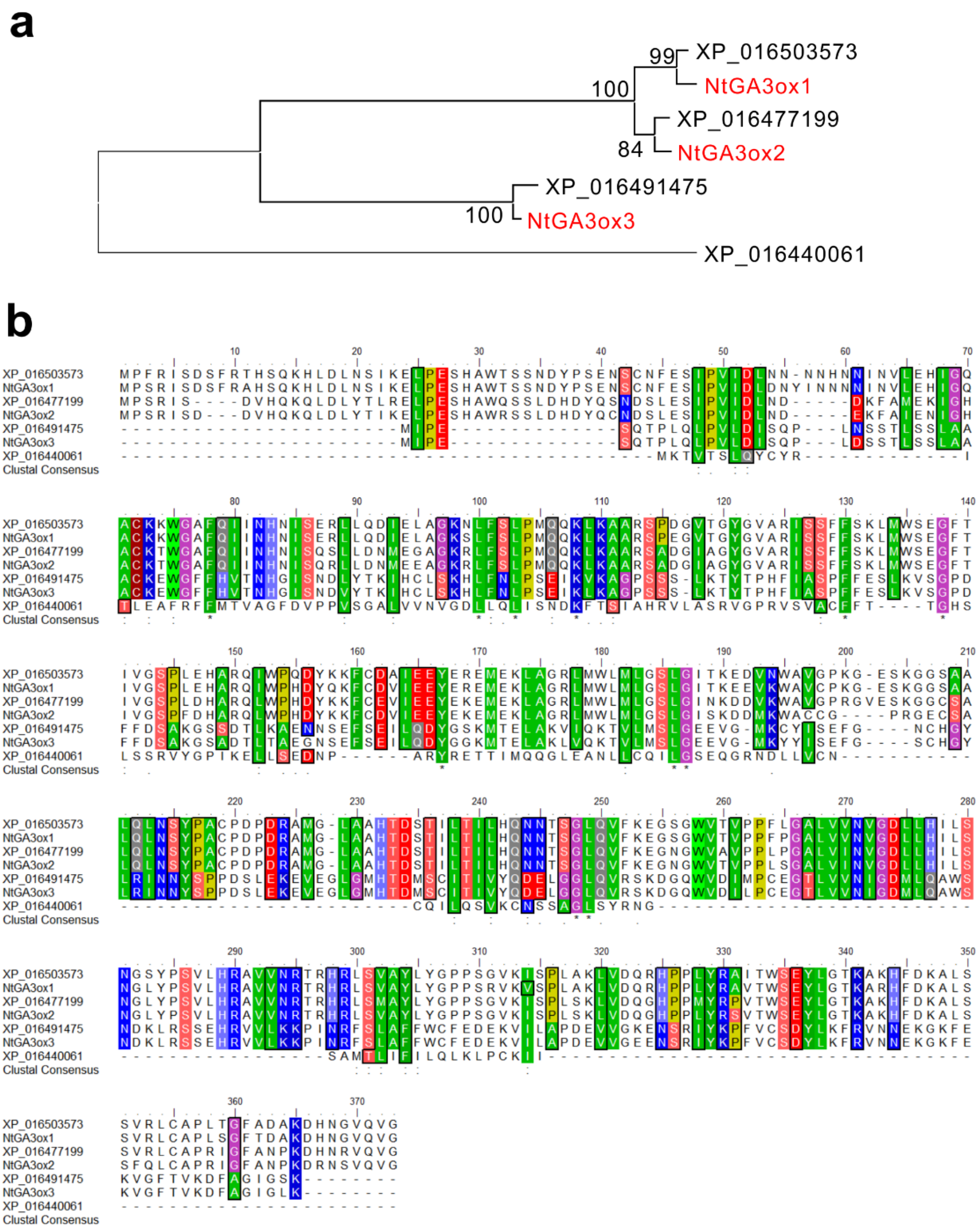

Supplementary Fig. 11: Phylogenetic analysis and sequence alignment of NtGA3ox proteins.

a Phylogenetic analysis of NtGA3ox and homologs proteins in *Nicotiana tabacum*. **b** A bootstrap consensus of phylogenetic tree representing similarities of NtGA3ox protein sequence.

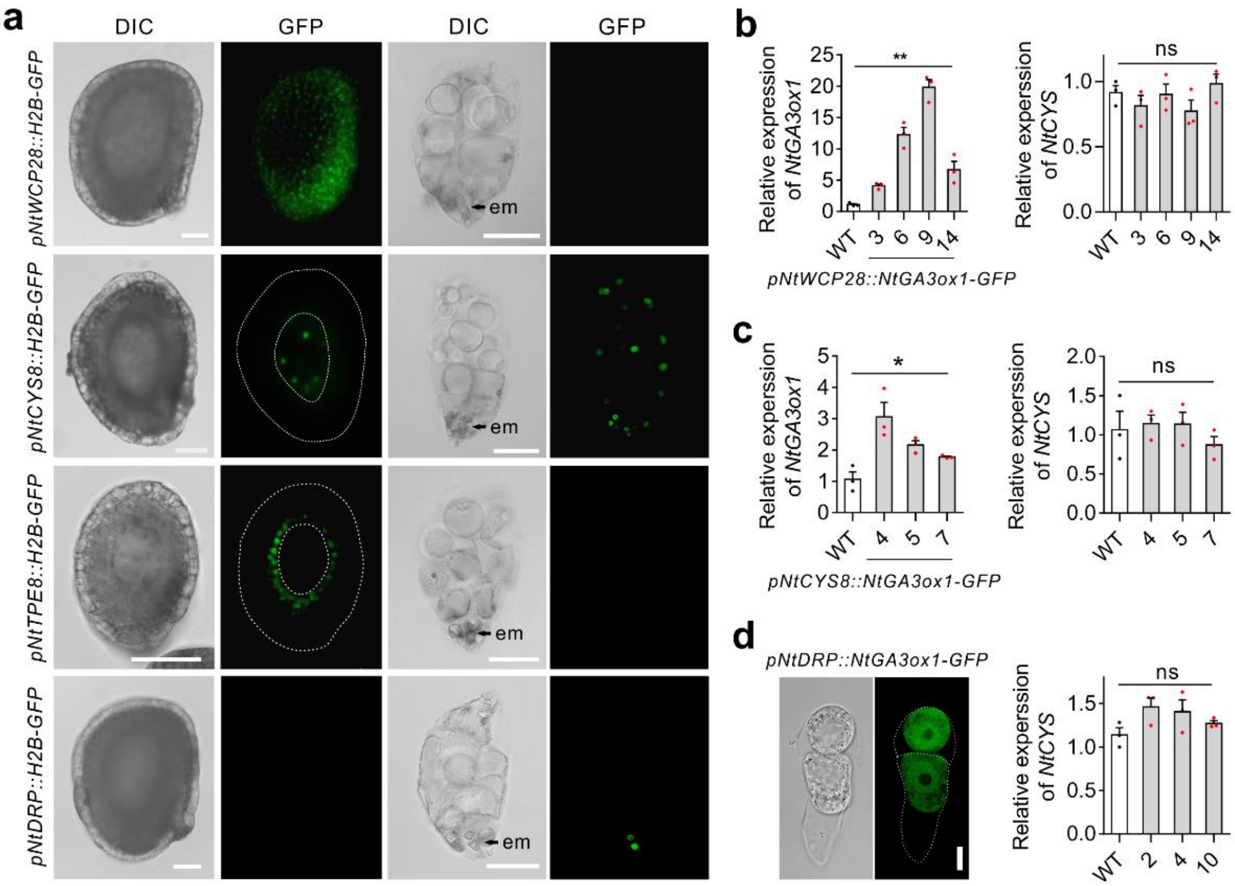

Supplementary Fig. 12: Integument-, endosperm- and embryo-specific expression of *NtGA3ox1-GFP* did not influence *NtCYS* expression.

a Promoter specificity of *NtWCP28* (*pNtWCP28::H2B-GFP* expression), *NtCYS8* (*pNtCYS8::H2B-GFP* expression), *NtTPE8* (*pNtTPE8::H2B-GFP* expression) and *NtDRP* (*pNtDRP::H2B-GFP* expression) in the seeds (left) and in the fertilized embryo sac with a proembryo (arrow) and endosperm cells (right). **b** Relative expression of *NtGA3ox1* and *NtCYS* in WT and *pNtWCP28::NtGA3ox1-GFP* lines. **c** Relative expression of *NtGA3ox1* and *NtCYS* in WT and *pNtCYS8::NtGA3ox1-GFP* lines. **d** *NtGA3ox1-GFP* expressed in proembryo and relative expression of *NtCYS* in WT and *pNtDRP::NtGA3ox1-GFP* lines. The expression level in WT was set to 1. Data represent the mean ± SE from three independent experiments. (Student's t-test, ns, $P > 0.05$, $^*P < 0.05$, and $^{**}P < 0.01$). Scale bars: 100 μm (**a**), 10 μm (**d**). The source data of the graphs are provided in the Source Data file.

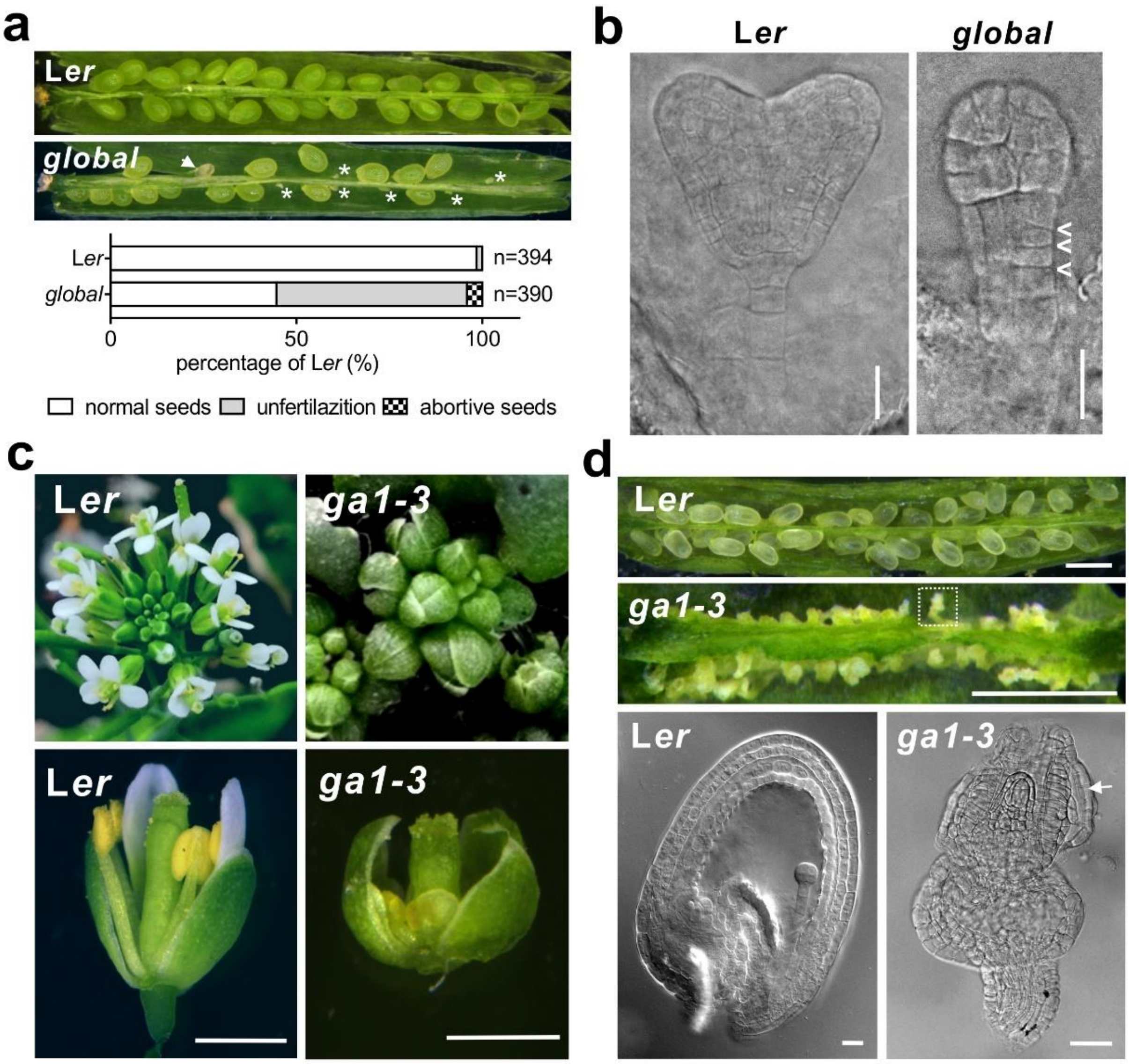

Supplementary Fig. 13: The phenotypes in *global-della* and *ga1-3* mutants in Arabidopsis. **a** Abortive seeds and reduced fertility in Arabidopsis *global-della* mutants. Arrow indicates abortive seeds. Stars indicate unfertilized ovules. **b** The proembryo of *global-della* mutant at the same stage. Arrows indicate extra cell divisions in an abnormal suspensor. **c** Arabidopsis *ga1-3* mutants show abnormal inflorescences and pistil. **d** Ovules of 48HAP WT (L*er*) and *ga1-3* plants. Arrow indicates arrested ovule with aborted female gametophyte development (98%, n=350). Scale Bar: 20μm (**b**) and (**d**) bottom; 0.5mm (**d**) top; 1mm (**c**).

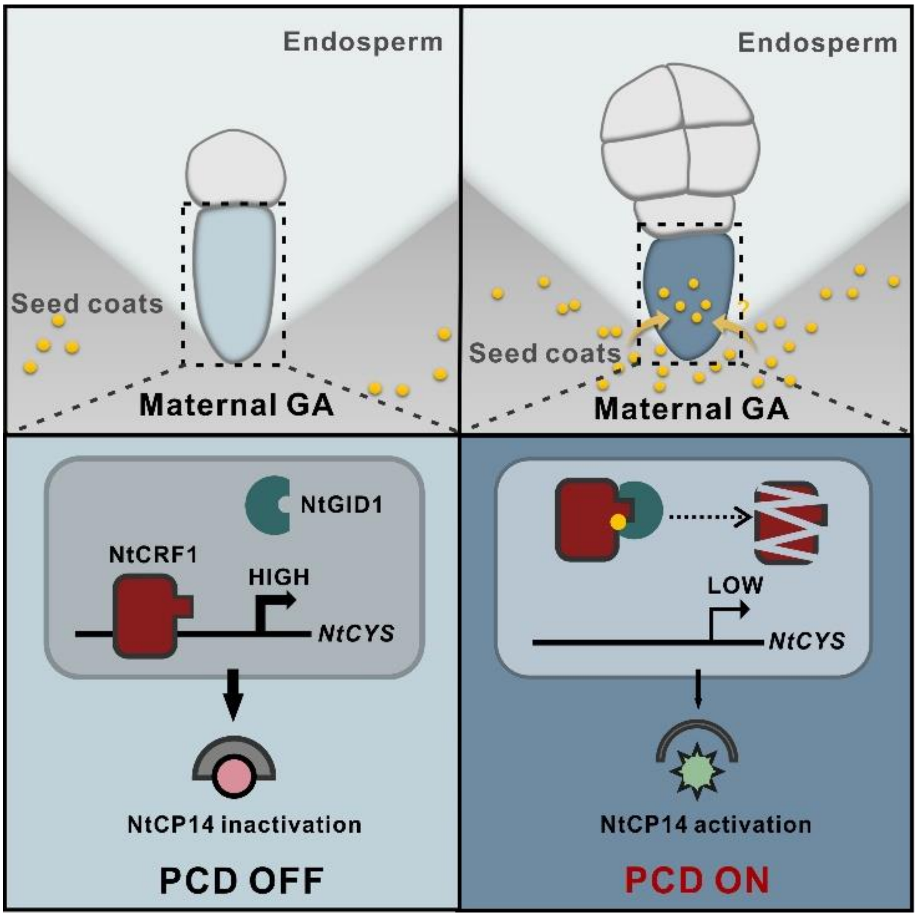

Supplementary Fig. 14: The work model of suspensor PCD.

Maternal-embryo communication via gibberellin signaling precisely controls the duration of maternal nursing of proembryos.
