# Supplementary material for: Maternal control of suspensor programmed cell death via gibberellin signaling
Source: Nat Commun. 2019 Aug 2;10:3484. doi: 10.1038/s41467-019-11476-3 (PMC6677759; doi:10.1038/s41467-019-11476-3)
Supplement: Supplementary file 2 — Description of Additional Supplementary Files [file 41467_2019_11476_MOESM2_ESM.pdf]

Description of Additional Supplementary Files

File Name: Supplementary Data 1
Description: The primers in the study.
